# Transcriptome sequencing and multi-plex imaging of prostate cancer microenvironment reveals a dominant role for monocytic cells in progression

**DOI:** 10.1186/s12885-021-08529-6

**Published:** 2021-07-22

**Authors:** Stefano Mangiola, Patrick McCoy, Martin Modrak, Fernando Souza-Fonseca-Guimaraes, Daniel Blashki, Ryan Stuchbery, Simon P. Keam, Michael Kerger, Ken Chow, Chayanica Nasa, Melanie Le Page, Natalie Lister, Simon Monard, Justin Peters, Phil Dundee, Scott G. Williams, Anthony J. Costello, Paul J. Neeson, Bhupinder Pal, Nicholas D. Huntington, Niall M. Corcoran, Anthony T. Papenfuss, Christopher M. Hovens

**Affiliations:** 1 Bioinformatics Division, The Walter and Eliza Hall Institute of Medical Research, Parkville, Victoria Australia; 2 Department of Surgery, The University of Melbourne, Parkville, Victoria Australia; 3 Department of Urology, Royal Melbourne Hospital, Parkville, Victoria Australia; 4 Department of Medical Biology, University of Melbourne, Melbourne, Victoria Australia; 5 Institute of Microbiology of the Czech Academy of Sciences, Prague, Czech Republic; 6 University of Queensland Diamantina Institute, Translational Research Institute, University of Queensland, Brisbane, QLD Australia; 7 The Peter Doherty Institute for Infection and Immunity, Parkville, Victoria Australia; 8 Sir Peter MacCallum Department of Oncology, University of Melbourne, Melbourne, Victoria Australia; 9 Peter MacCallum Cancer Centre, Melbourne, VIC 3000 Australia; 10 Flow Cytometry Facility, The Walter and Eliza Hall Institute of Medical Research, Parkville, Victoria Australia; 11 Cancer Program, Biomedicine Discovery Institute, Monash University, Clayton, Victoria Australia; 12 Department of Anatomy and Developmental Biology, Monash University, Clayton, Victoria Australia; 13 Epworth Center of Cancer Research, Clayton, Victoria Australia; 14 The Olivia Newton-John Cancer Research Institute, Heidelberg, Melbourne, Australia; 15 Department of Urology, Frankston Hospital, Frankston, Victoria Australia; 16 School of Mathematics and Statistics, University of Melbourne, Melbourne, VIC 3010 Australia

**Keywords:** Prostate cancer, Transcriptomics, FACS, Immunohistochemistry, Deconvolution, Bayes, Differential gene expression, CAPRA-S, Microenvironment, Epithelial, Myeloid, Macrophages, Cholesterol, PDL1

## Abstract

**Background:**

Prostate cancer is caused by genomic aberrations in normal epithelial cells, however clinical translation of findings from analyses of cancer cells alone has been very limited. A deeper understanding of the tumour microenvironment is needed to identify the key drivers of disease progression and reveal novel therapeutic opportunities.

**Results:**

In this study, the experimental enrichment of selected cell-types, the development of a Bayesian inference model for continuous differential transcript abundance, and multiplex immunohistochemistry permitted us to define the transcriptional landscape of the prostate cancer microenvironment along the disease progression axis. An important role of monocytes and macrophages in prostate cancer progression and disease recurrence was uncovered, supported by both transcriptional landscape findings and by differential tissue composition analyses. These findings were corroborated and validated by spatial analyses at the single-cell level using multiplex immunohistochemistry.

**Conclusions:**

This study advances our knowledge concerning the role of monocyte-derived recruitment in primary prostate cancer, and supports their key role in disease progression, patient survival and prostate microenvironment immune modulation.

**Supplementary Information:**

The online version contains supplementary material available at 10.1186/s12885-021-08529-6.

## Background

Prostate cancer is the second most commonly diagnosed cancer in men globally [1]. Although most cancers follow an indolent clinical course, an unpredictable 10–15% of tumours progress to metastases and death. The inability to discern progressive disease at an early stage results in substantial overtreatment of localised disease, leading to a significant clinical cost to the patient and economic cost to the healthcare system. Selecting patients for treatment is usually reliant on a small number of well-established clinical and pathological factors, such as tumour grade, prostate serum antigen (PSA) level and clinical stage [2], the development of metastases [3] and prostate cancer-specific death [4]. Although comprehensive molecular analyses have linked clinical outcomes with rates of genomic alterations [5, 6], such as somatic changes in copy number, nucleotide sequence and methylation, it is yet to be demonstrated that such measures are able to consistently outperform standard clinico-pathological risk scoring across a broad range of grades and stages. Despite many years of tumour evolution characterization, it still remains unclear what mechanisms drive prostate cancer progression in most patients [7]. It is believed that reciprocal interactions between malignant epithelium and surrounding non-cancerous cells within the tumour microenvironment are responsible for driving disease progression [8, 9].

Selected targets in the prostate tumour microenvironment have been extensively studied through in vitro and in vivo experiments, such as migration assays [10] and xenograft mouse models [11] respectively. More recently, several studies that integrated fluorescence-activated cell sorting or laser microdissection with RNA sequencing increased the gene and sample throughput while maintaining a degree of resolution of the tissue heterogeneity [8, 12, 13]. Additionally, the use of spatial transcriptomics has identified gradients of benign-cell gene transcription around tumour foci [14]. However, these studies mainly focused on the process of epithelial to mesenchymal transition [12, 13] or were limited to the overall stromal contribution to disease progression [8]. An integrative investigation of immune, stromal and cancer cell transcriptional changes associated with clinical risk is still lacking.

In this study, we applied an optimised protocol for combined cell-type enrichment and ultra-low-input RNA sequencing, which allowed the probing of four key cell types across 13 fresh prostate tissue spanning a broad clinical disease spectrum. Motivated by the pseudo-continuous properties of the CAPRA-S risk score, we developed a novel statistical inference model for differential transcription analyses on continuous covariates, TABI (Transcriptional Analysis through Bayesian Inference). Our inference model estimated changes in transcription without a priori patient risk stratification and robustly mapped transcriptional change events to cancer risk states. Among the list of significant genes for the four cell types coding for cell-surface and secreted proteins, we identified several hallmarks of prostate cancer. These hallmarks included a dominant signal for monocyte-derived cell recruitment. We tested this with tissue deconvolution on the extensive Cancer Genome Atlas (TCGA) cohort and multiplex immunohistochemistry on an independent patient cohort. The latter single-cell resolution spatial analysis revealed the relationship between macrophages and epithelial and T cells with progression. For prostate cancer, prioritising targets for immunotherapies is far from settled in the literature, with monocytic cells being an under-represented player [15, 16]. In this scenario, the parallel lines of evidence we provide from unbiased analyses contribute to shaping future research directions.

## Methods

### Tissue sampling and processing

Following the prostatectomy of 13 patients, a four-millimetre tissue core was collected from the prostate tumour site, conditional to histopathological verification [17, 18]. The patient cohort ranged from 52 to 78 years of age and from CAPRA-S risk score of 0 (attributed to benign tissue samples, harvested from a site far from a low grade, low volume cancer) to 7 (Supplementary file 4), If not otherwise specified, all procedures were carried out at 4 °C. Tissue blocks were washed in phosphate-buffered saline (PBS) solution for 2 min and minced for 2 min with a scalpel. Homogenised tissue was added to a solution (total volume of 7 ml) composed by of 1 mg/ml collagenase IV (Worthington Biochemical Corp, USA), 0.02 mg/ml DNase 1 (New England Biolabs, USA), 0.2 mg/ml dispase (Merck, USA). The homogenised tissue was serially digested in the shaker incubator at 37 °C at 180 rpm (4 g), through three steps of 5, 10 and 10 min of duration. The final 3 min were dedicated to sedimentation at 0 rpm. After each digestion step, the supernatant was aspirated and filtered through a 70 μm strainer into a pre-chilled tube, diluting the solution with 15 ml of Dulbecco’s PBS containing 2% Bovine serum (dPBS-serum) to quench the enzymatic reaction. The resulting cumulative solution was then centrifuged at 300gfor five minutes, with the supernatant collected and the cell pellet resuspended into 1 ml 2% PBS-serum before labelling (Fig. S1).

### Antibody labelling, flow cytometry and cell storage

The cell preparation was labelled with the following antibodies: CD3-BV711 (Becton Dickinson San Jose Ca), EpCAM-PE (BD Biosciences, USA), CD31-APC (BD Biosciences, USA), CD90-PerCP-Cy5.5 (Becton Dickinson San Jose Ca), CD45 APC-CY7, and CD16 Pacific Blue (BD Biosciences, USA). All antibodies were used at concentrations according to manufacturers recommendations and incubated for 30 mins at 4 °C. Following labelling, the cells were diluted to 5 ml and centrifuged at 300 g for 5 min. The supernatant was removed, and the cell pellet was resuspended in dPBS-serum. The viability die (7AAD) was added to the suspension to a final concentration of 5 μg/ml. Epithelial, fibroblasts, myeloid and T cells were sorted using a fluorescence-activated cell sorting Aria III cell sorter (Becton Dickinson San Jose, Ca). The cell sorting strategy utilised a robust 3 stage design: (i) a series of gates based on forward and side scatter to exclude debris, cell clumps and doublets. (ii) a gate to exclude all dead cells and (iii) a combination of the fluorescent antibodies to allow purification of the above cell types. The four cell types were identified as follows: T Cells: FSC and SSC lo, PI negative, EpCAM and CD31 negative, CD3 and CD45 positive. Epithelial cells: FSC and SSC high, PI negative, CD31 and CD90 negative and EpCAM positive. Myeloid cells: FSC and SSC hi and medium, PI negative, CD31 and EpCAM negative and CD16 positive. Fibroblasts: FSC and SSC hi, PI negative, EpCAM and CD31 negative, CD90 positive. The four purified populations were sorted directly into 1.5 ml conical tubes and stored on dry ice immediately after collection before permanent storage at − 80 °C.

### RNA extraction, library preparation and RNA sequencing

RNA extraction was performed in two batches (comprising 6 and 7 patients, for a total of 24 and 28 samples, respectively) on consecutive days. In order to eliminate time-dependent methodological biases, the two patient batches included a balanced distribution of Gleason score (means 2.00 and 2.71, standard deviations 2.50, 1.86; Supplementary file 4) and days elapsed from tissue processing (means 197 and 222, standard deviations 46.3 and 71.9; Supplementary file 4). The RNA extraction was performed using the miRNeasy Micro Kit (Qiagen; Cat #217084), according to the manufacturer’s protocol. Briefly, cell pellets were lysed with QIAzol lysis reagent, treated with chloroform, and centrifugation carried out to separate the aqueous phase. Total RNA was precipitated from the aqueous phase using absolute ethanol, filtered through the MinElute spin column and treated with DNase I to remove genomic DNA. The RNA bound columns were washed with the buffers RWT and RPE before eluting the total RNA with 14 μl of RNase-free water. RNA estimation was carried out using Tapestation (Agilent).

According to the manufacturer’s protocol, transcriptome sequencing on low input total RNA samples (up to 10 ng) was carried out using SMART-Seq v4 Ultra Low Input RNA Kit (Clontech). The first-strand cDNA synthesis utilised 3′ SMART-Seq CDS Primer II-A. The SMART-Seq v4 Oligonucleotide together with the cDNA amplification was carried out on Thermocycler using PCR Primer II-A and PCR conditions: 95 °C for 1 min, 12 cycles of 98 °C 10 s, 65 °C 30 s and 68 °C 3 min; 72 °C for 10 min and 4 °C until completion. The PCR-amplified cDNA was purified using AMPure XP beads and processed with the Nextera XT DNA Library Preparation Kits (Illumina, Cat. # FC-131-1024 and FC- 131-1096) as per the protocol provided by the manufacturer.

Sequencing library preparation (10–100 ng) was carried out using Truseq RNA Sample Preparation Kit v2. The poly-A containing mRNA was purified using oligo-dT bound magnetic beads followed by fragmentation. The first-strand cDNA synthesis utilised random primers, and second-strand cDNA synthesis was carried out using DNA Polymerase I. The cDNA fragments then underwent an end-repair process, adding a single ‘A’ base and ligation of the RNA adapters. The adaptor-ligated cDNA samples were bead-purified and enriched with PCR (15 cycles) to generate the final RNAseq library.

The SMART-Seq v4 RNA and Truseq RNA libraries were sequenced on an Illumina Nextseq 500 to generate 15–20 million 75 base pairs paired-end reads for each sample. The batch effect due to sequencing runs was minimised by pooling all 52 libraries and carrying out three sequential runs on a Nextseq500 sequencer.

### Sequencing data quality control, mapping and read counting

The quality of the sequenced reads for each sample was checked using the Fastqc [19]. Reads were trimmed for custom Nextera Illumina adapters; low-quality fragments and short reads were filtered out from the pools using BBDuk (jgi.doe.gov) according to default settings. All remaining reads were aligned to the reference genome hg38 using the STAR aligner [20] with default settings. The quality control on the alignment was performed with RNA-SeQC [21]. For each sample, the gene transcription abundance was quantified in terms of nucleotide reads per gene (read-count) using FeatureCounts [22] with the following settings: isPairedEnd = T, requireBothEndsMapped = T, checkFragLength = F, useMetaFeatures = T. All sequenced reads that did not align to the reference human genome were assigned to bacterial and viral reference genomes using Kraken [23] with default settings.

### Statistical inference of differential gene transcript-abundance

Changes of transcriptional levels along CAPRA-S risk score [24] were estimated independently for each cell type (epithelial, fibroblast, myeloid and T cell). The CAPRA-S risk score is a combination of (i) concentration of blood prostate serum antigen (PSA); (ii) presence of surgical margin (SM); (iii) Gleason score; (iv) presence of seminal vesicle invasion (SVI); (v) the extent of extracapsular extension (ECE); and (vi) lymph node involvement. The RNA extraction batch was used as a further covariate. Due to the absence of publicly available models for non-linear monotonic regression along a continuous covariate, a new Bayesian inference model was implemented. This model is based on the simplified Richard’s curve [25] (Eq.1) but re-parameterised to improve numerical stability (Eq. 2). In particular, the standard parametrisation suffers from non-determinability issues if the slope is close to zero; furthermore, in the case of an exponential-like trend, the upper plateau is not supported by data and tends to infinity.

1 $$ GL\;\left(X,\alpha, \beta, \kappa \right)=\frac{k}{1+{e}^{-\left(\alpha + X\beta \right)}} $$

2 $$ GLA\;\left(X,{y}_0,\beta, \eta \right)=\frac{y_0\left(1+{e}^{{\eta \beta}_1}\right)}{1+{e}^{{\eta \beta}_1- X\beta}} $$

The new parameter y_0_ represents the intercept on the y axis, η represents the point of inflection on the x-axis, β represents the matrix of coefficients (i.e. slope coefficients, without the intercept term), β_1_ represents the coefficient of interest (i.e. main slope), and k the upper plateau of the generalised sigmoid function.

Bayesian inference was used to infer the values of all parameters of the model, with TABI (GitHub: stemangiola/TABI@v0.1.3). The probabilistic framework Stan [26] was used to encode the joint probability function of the model (Eq. 3). We partitioned the transcriptomic dataset into blocks of 5000 genes to decrease the analysis run-time. This Bayes model is based on a negative binomial distribution (parameterised as mean and overdispersion). In order to account for various sequencing depths across samples, a sample-wise normalisation parameter was added to the negative binomial expected value. The slope parameter for the main covariate (β_1_) was subject to a regularised horseshoe prior [27] to increase the robustness of the inference of transcription changes and help anchor data from different samples for normalisation. The role of this prior is to impose a sparsity assumption on the gene-wise transcriptional changes; that is, most genes are not Tdifferentially transcribed. The overall distribution of the gene intercepts follows a gamma probability function. The following joint probability density defines the statistical model.

3 $$ {\displaystyle \begin{array}{l}P\left(\gamma \right)P\left(\delta \right)P\left(\sigma \right)P\left(\eta \right)P\left(\xi \right)P\left(\dot{\beta}\left|\xi \right.\right)\\ {}\left(\prod \limits_{r=2}^RP\left({\beta}_r\left|\sigma \right.\right)\right)\left(\prod \limits_{g=1}^GP\left({y}_{\mathrm{o}g}\left|{\gamma}^{\prime },{\gamma}^{{\prime\prime}}\right.\right)\right)\\ {}\left(\prod \limits_{g=1}^G\prod \limits_{s=1}^SP\left({Y}_{g,s}\left|\hat{Y},\delta, \omega \right.\right)\right)\end{array}} $$

4 $$ {Y}_{t,g}\sim NB\;\left(\mathit{\exp}\left({\delta}_t\right){\hat{Y}}_{t,g},\omega \right) $$

5 $$ {\hat{Y}}_{t,g}= GLA\left({X}_{t,}{y}_{0_g},{\beta}_g,{\eta}_g\right) $$

6 $$ {\beta}_{g,1}\sim \mathit{\operatorname{Re}} gHorseshoe\left(\dots \right) $$

7 $$ {\displaystyle \begin{array}{l}{\beta}_{g,k}\sim N\left(0,{\sigma}_k\right);k>1\\ {}{\sigma}_k\sim HalfN\left(0,1\right)\end{array}} $$

8 $$ {\displaystyle \begin{array}{l}{y}_{0_g}\sim Gamma\left({\gamma}_1+1,{\gamma}_2\right)\\ {}{\gamma}_i\sim Exponential(1)\\ {}\omega \sim Gamma\left(1.02,2\right)\end{array}} $$

9 $$ {\displaystyle \begin{array}{l}{\eta}_g\sim N\left(0,1\right)\\ {}{\delta}_t\sim N\left(0,1\right);\sum {\delta}_t\sim N\left(0,0.001\ast T\right)\end{array}} $$

Y represents raw transcript abundance, $\underset{\_}{\hat{Y}}$ represents the expected values of transcript abundance, and X represents the design matrix (with no intercept term and scaled covariates). The regression function also includes β, which represents the gene-wise matrix of factors (i.e. slopes excluding the intercept term), $\underset{\_}{y}$ and η, which represent the gene-wise y-intercept and the inflection point of the generalised reparameterised sigmoid function (Eq. 2). γ represents the hyperparameters of $\underset{\_}{y}$. Other parameters of the negative binomial function are δ, which represents the normalisation factors, and ω, which represents overdispersion. The regularising prior (for imposing the sparsity assumption) over the covariate of interest β_1_ (first column of β) is defined by the hyperparameter list ξ [27] (i.e. nu_local = 1; nu_global = 1; par_ratio = 0.8; slab_df = 4; slab_scale = 0.5), while σ represents the standard deviations of the other factors (in our case only the batch). The algorithm multidimensional scaling [28] was used to map the data in two-dimensional space.

### Gene annotation

Each gene (g) was considered well fitted by the model if it had read counts outside the 95th percentile of the generated quantities for three or fewer samples (according to posterior predictive checks standards [29]). Among the well-fitted genes, those for which the 0.95 credible intervals of the posterior distribution of the factor of interest β_1g_ did not include the value 0 were labelled as differentially transcribed. The credible interval is a numerical range within which an unobserved parameter value falls within a certain probability. As distinct from standard practices for frequentist models operating on confidence intervals and *p*-values, for this study, the credible interval probability threshold was not altered for multiple hypothesis testing, consistently with standard practices in Bayesian statistics [30].

In order to interpret the inflection points over the CAPRA-S risk score (i.e. the point of the maximum slope; at what stage of the disease a transcriptional change happens) covariate in a biologically meaningful way, the inflection point was adjusted to the log-scale. Considering that the lower plateau of our generalised sigmoid function was set to 0 (to limit the number of parameters needed to model it), the inflection point of the logarithm-transformed function is not defined. Therefore, we calculated the inflection point (*X*) of the log sigmoid forcing a plateau at 1 (i.e. log (0) = 1; Eq. 10; Fig. S7). This new inflection point can now be calculated as the value of the x-axis at half distance between zero and the upper plateau of the generalised reparameterised sigmoid function (Eq. 10).

10 $$ \dot{X}=\frac{\beta_1\eta -\mathit{\log}\left({e}^{\frac{y0}{2}}\sqrt{e^{y0\eta }+1-1}\right)}{y0} $$

Genes were functionally annotated with gene ontology categories [31] using BiomaRt [32]. Furthermore, genes were functionally annotated with the protein atlas database [33] for identifying those that interface with the extracellular environment, encoding for cell-surface and secreted proteins. For a more in-depth analysis of possible interactions between cell types, we compiled a cell-type-specific annotation database for cell-surface and secreted protein-coding genes (Supplementary file 3).

### Differential tissue composition analyses

The differential tissue composition analysis is composed of two integrated modules. First, a module infers tissue composition from whole-tissue gene transcript abundances based on reference transcriptional profiles of pure cell types (deconvolution). Second, a module for beta regression on the inferred proportions along the factor of interest (and additional covariates). Bayesian inference allows the transfer of the uncertainty between the two modules (GitHub: stemangiola/ARMET@v0.7.1). The probabilistic framework Stan [26] was used to encode the joint probability function of the model [34]. The 0.95 credible interval of the posterior distributions was used as a significance threshold.

The supervised deconvolution was based on deconvolution signatures created using a curated collection of 250 publicly available transcriptional profiles (included in BLUEPRINT [35], ENCODE [36], GSE89442 [37] and GSE107011 [38]) encompassing of 8 broad categories of cell types and 18 cell phenotypes. Genes whose transcription varied across datasets (detected using Limma [28]) were used to identify highly correlated datasets. The Pearson correlation was calculated for all-versus-all samples. The samples with a Pearson correlation greater than 0.99 were discarded as redundant. Each cell-type category was classified as belonging to a node of the cell-differentiation tree, which includes epithelial, fibroblasts, endothelial and immune cells in the first level, and B-, T-, natural killer, monocyte-derived, and granulocyte cells. For each cell type in the differentiation tree, the gene-transcript abundance was modelled using a negative binomial distribution (parameterised by mean and overdispersion). Differences in sequencing depth across biological replicates were modelled with a biological replicate-wise exposure rate term ϵ that multiplies the transcripts expected abundance (mean). For each cell-type pair of the same level, 40 genes (20 for each direction) were selected that (i) were abundant (had a mean value higher than the median of all genes), and (ii) segregated the two cell types (having the largest gap between the upper quantile of one cell-type and the lower quantile of the other; 95% credible interval). The gene selection for each level was represented by the union of marker genes for all cell-type pairs. The inference was carried out along the two levels of the hierarchy structure, and the inference for each node (e.g. T-cells) was relative to its parent (e.g. immune cells).

### Analysis of tumour microenvironment using multiplex immunohistochemistry

Slides (3 μm) from formalin-fixed and paraffin-embedded (FFPE) tissue were taken from a total of 63 core biopsies of localised prostate cancer across 17 patients. A pathological evaluation was done to define the tumour and surrounding benign tissue areas for each biopsy. The methodology for performing multiplex immunohistochemistry, cell type classification and localisation has been detailed by Keam et al. [39]. Briefly, slides were deparaffinised and rehydrated with xylene and ethanol. The fluorochrome-coupled antibodies against human CD68 (macrophages and dendritic cells), high molecular weight cytokeratin (HMWCK; epithelial basal cells), CD3 (T cells), CD20 (B cells), CD11c (dendritic cells), and PDL1 were used. The dye DAPI was used for nuclei staining. Vectra 3.0 Automated Quantitative Pathology Imaging System (Perkin Elmer, MA) was used for imaging, as Keam et al. [39] detailed. The software HALO was used for cell segmentation and phenotyping. Stromal cells were defined with the negative selection of all antibodies (DAPI positive) and with filtering by large size (cell area > 70) and highly elongated shape (ratio of largest dimension and smallest dimension > 2; 0.9 percentile; 0.9 percentile; Fig. S6).

Cell type proximity was quantified as the number of cells within a radius of 20 cells sizes from a selected cell, averaged per tissue area (5 cell size units) for smoothing and avoiding information duplication due to tight cell clusters. Cell relative size was calculated at 15 units as the observed median length units in the coordinate system. The statistics were summarised at the biopsy level. When the distance between two cell types was measured, only the biopsies including both cell types were selected. The robust regression analyses were performed using the R heavy package [40] on log-transformed proximity measure. The co-proximity analysis between epithelial basal cells and PDL1+ macrophages and T cells was performed at the single cell level (averaged by tissue area of 5 cell size units). We calculated the proximity on a radius of 50 relative cell sizes for ensuring good coverage of both T cells and PDL1+ macrophages and decrease sparsity. Only the epithelial basal cells in immune rich areas (with > 5 neighbour T cells) were considered.

## Result

### Data generation and quality

To investigate the role of the tumour microenvironment in patient outcome, we enriched for four cell populations (epithelial: EpCAM^+^; fibroblasts CD90^+^/CD31^−^; T cells: CD45^+^/CD3^+^; and myeloid: CD45^+^/CD16^+^) from fresh prostatectomies of 13 prostate cancers, ranging from benign tissue (labelled as CAPRA-S score 0) to high-risk tumours (CAPRA-S risk score 7). The choice of those cell populations was guided by their predominant role in the progression of prostate and other cancers [41–45]. Technical and practical experimental limitations prevented considering other key cell types such as luminal and basal epithelial compartments, endothelial, smooth muscle and other lymphocytes such as B and natural killer cells. RNA extracted from the four cell populations was then sequenced, generating a median of 22 million reads per library (Fig. S1 and S2). Overall for the four cell type categories, a conservative cell-type purity inference (using Cibersort and a Bayesian estimator; Material and Methods) estimated high enrichment: 99% for epithelial samples; 99% for fibroblasts; 97% for myeloid (83% for neutrophils; 14% for monocyte-derived cells); and 95% for T cells (Fig. S3). On average, across the four cell types, 40% of genes had 0 sequenced reads in more than half of the samples and were removed from further analysis.

### Differential transcription and model fitting

**Fig. 1:**
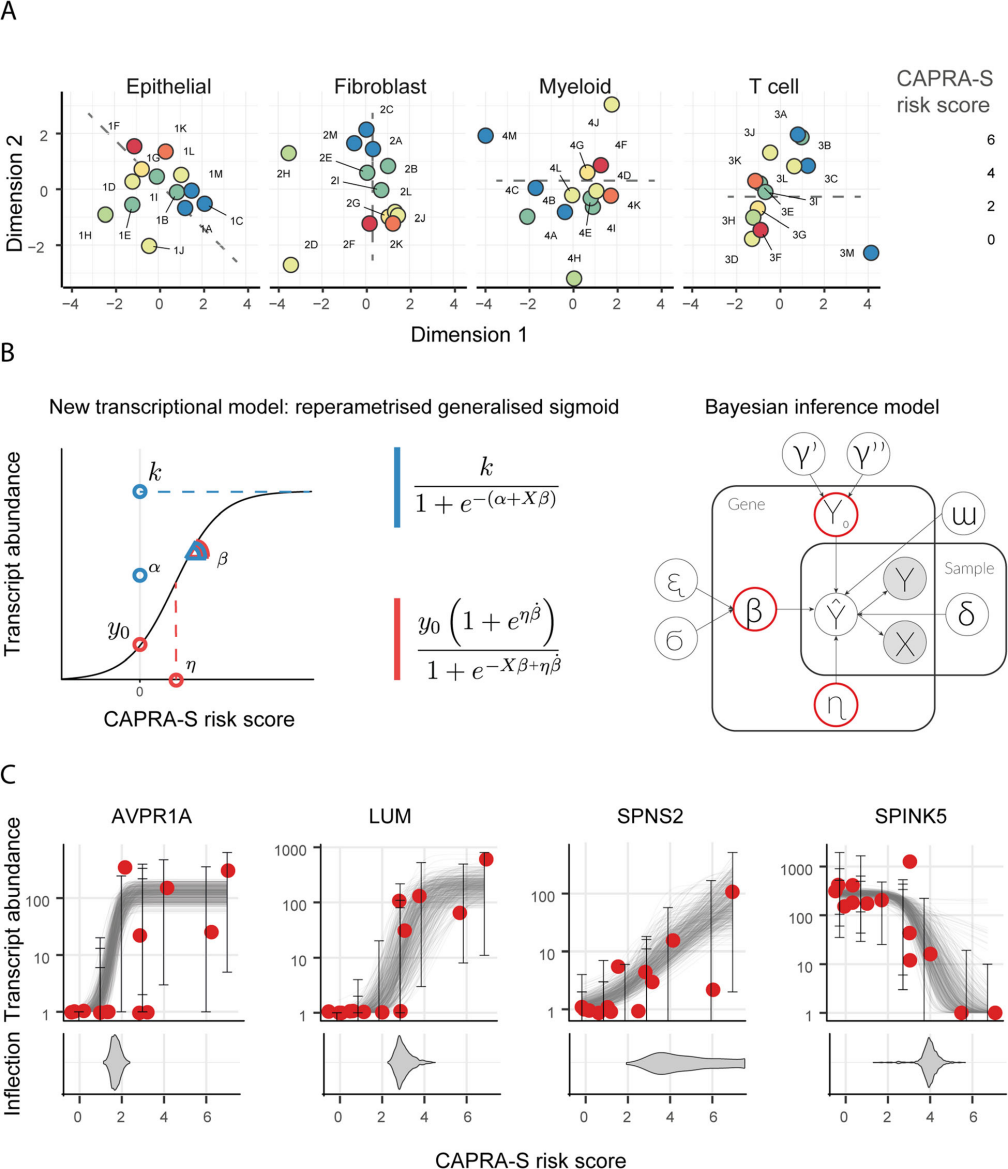
The continuous relationship between the CAPRA-S risk score and gene transcript abundance. **A** Multidimensional scaling plots of transcript abundance grouped by cell type. The colour coding represents the CAPRA-S risk score. The risk-score is correlated with the first and second dimension, particularly in epithelial and fibroblast cells (linear regression performed using lm in R; Bonferroni adjusted *p*-value of 0.0187, 0.00971, 0.0306 and 0.367, respectively). Alphanumeric codes refer to patient identifiers (Supplementary Table S1). The dashed lines indicate the correlation between the first and the second dimension with the CAPRA-S risk score. **B** Re-parameterisation of the generalised sigmoid function and probabilistic model (Material and Methods). Left-panel: The three reference parameters for the standard parameterisation (blue). Alternative robust parameterisation (red). Right-panel: a graphic representation of the probabilistic model TABI. **C** Examples of continuous relationships between transcript abundance of four representative genes and CAPRA-S risk score (for epithelial cell population), from more discrete-like to more linear-like. The bottom panel displays the inferred distribution of possible values (as posterior distribution) of the inflection point for each gene sigmoid trend

Dimensionality reduction (multidimensional scaling; in Materials and Methods) of the filtered transcript abundance revealed an association of CAPRA-S risk score with either the first and the second principal components (Fig. 1a; with an indicative direction represented by the dashed grey line; tested with linear regression, lm function from R). A clear gradient in risk score was seen for epithelial and fibroblasts (Bonferroni adjusted *p*-value of 1.0 × 10^− 2^ and 9.7 × 10^–3,^ respectively). A weaker pattern was apparent for myeloid and T cells (Bonferroni adjusted p-value of 3.0 × 10^− 2^ and 0.37 respectively), possibly due to the greater heterogeneity of the two immune cell populations than epithelial and fibroblasts.

We performed a differential transcript abundance analysis (at the gene level) for each cell type independently, seeking associations between transcript abundance across subjects and CAPRA-S risk score, treated as pseudo-continuous variables. In order to perform differential analyses that would robustly model the pseudo-continuous properties of the CAPRA-S risk score (Fig. 1a), we developed a Bayesian inference model (TABI) that implements a robust generalised sigmoid regression (i.e. sigmoid function extending from zero to any positive value). TABI was used to model the gene transcript abundance as a continuous function of CAPRA-S risk score (from 0 representing benign to 7 representing high risk); this avoids the loss of information caused by patients’ a priori stratification in low−/high-risk groups based on an arbitrary threshold. In principle, using a generalised sigmoid function permits modelling linear, exponential and sigmoid-like trends of transcriptional alterations (Fig. 1b and c). However, to provide robust modelling for RNA sequencing data, we reparameterised the generalised sigmoid function to suit better the numerical properties of transcript abundance (Fig. 1b; Materials and Methods). In addition to robustness, the sigmoid function allows mapping each differential transcriptional event with a clinical risk state, effectively providing a new developmental dimension to the analyses. This mapping is possible because the inflection point represents the CAPRA-S risk score at which the transcriptional alteration is most pronounced. The location of the most rapid change can be highly localised in the case of a dramatic change in transcription at a specific risk score or can be diffused in the case of a gradual change of transcription along the risk score range (Fig. 1c).

Following the statistical inference of transcriptional alterations, an average of 10% of genes was removed based on the posterior predictive check across all samples [29], as not satisfying the assumptions of our model (Table 1; Materials and Methods). A total of 1626 genes were identified as differentially transcribed across the four cell type categories (i.e. 95% credible interval excluding zero; with no need for multiple test adaptation, consistent with standard practices in Bayesian statistics [30]; Table 1; Supplementary file 1). The distributions of differential transcription events along the CAPRA-S risk score range are concentrated on low-risk scores (Fig. S4) for the four cell types, indicating that most transcriptional changes occur early in cancer developmental stages (including benign prostate tissue).

**Table 1:** Summary statistics of the differential transcription analysis, including 52 samples from 13 patients and 4 enriched cell types

Cell type	Total genes	Genes filtered (zeros)	Genes filtered (PPC)	Differentially transcribed			Differentially transcribed in the interface (curated annotation)		
				Total (up/down)	Of which cancer genes	Of which PC genes	Total (up/down)	Of which cancer genes, consistent	Of which PC genes, consistent
Epithelial	21,618	5408	189	171 (139/32)	45 (26%)	29 (64%)	80 (67/13)	35 (44%)	23 (67%)
Fibroblast	21,510	7141	651	267 (156/111)	27 (10%)	9 (33%)	97 (58/39)	17 (18%)	7 (41%)
Myeloid	22,507	13,836	2695	900 (827/73)	56 (6%)	11 (20%)	261 (238/23)	32 (12%)	10 (31%)
T cell	21,716	8807	540	288 (195/93)	42 (15%)	18 (42%)	83 (55/28)	26 (31%)	15 (58%)

*PPC* posterior predictive check, *PC* prostate cancer. “Of which” refers to the gene selection relative to the category adjacent on the left. “Interface” refers to cell-surface and secreted protein-coding genes. “Curated” refers to the curated database for cellular-interface genes produced in our study (Supplementary file 2). “Consistent” refers to a consistent direction of transcriptional change according to the curated database. Genes were labelled as “cancer genes” if present in the tier1 COSMIC databasehttps://paperpile.com/c/BQQ95X/zLPNs [46] or labelled as such in our manually curated cell-type-specific database (Supplementary file 2). Genes were labelled as “prostate cancer genes” if present in the tier1 COSMIC prostate cancer database datasethttps://paperpile.com/c/BQQ95X/zLPNs [46] or labelled as such in our manually curated cell-type-specific database (Supplementary file 2)

### Differentially transcribed cell-surface and secreted protein-coding genes are linked with recurring cancer hallmarks

In order to provide an initial biological evaluation of the resultant differentially transcribed genes, we sought the overlap with cancer-related gene datasets and calculated the enrichment of gene sets against functional and clinical gene annotation databases. On average, across the four cell types, 14% of all the differentially transcribed genes have been previously identified as cancer-related; of these, 24% have been previously described as prostate cancer-related genes [46] (Table 1). For differentially transcribed cell-surface and secreted protein-coding genes, an average of 33 and 51% have been previously described as cancer and prostate cancer-related genes, respectively [46] (Table 1). In order to investigate possible cell-cell interactions within the primary prostate tumour microenvironment, we focused on genes encoding for cell-surface and secreted proteins, which may directly influence other cell types. On average, across the four cell types, 35% of differentially transcribed genes encode for cellular-interface proteins; of those, 148 genes have been previously described as cancer-related genes. For all cell types, most cancer genes have a direction of change for all cell types consistent with the direction reported in the literature (35 vs 13 for epithelial; 17 vs 8 for fibroblasts; 32 vs 6 for myeloid cells; and 26 vs 11 for T cells; Supplementary file 3).

**Fig. 2:**
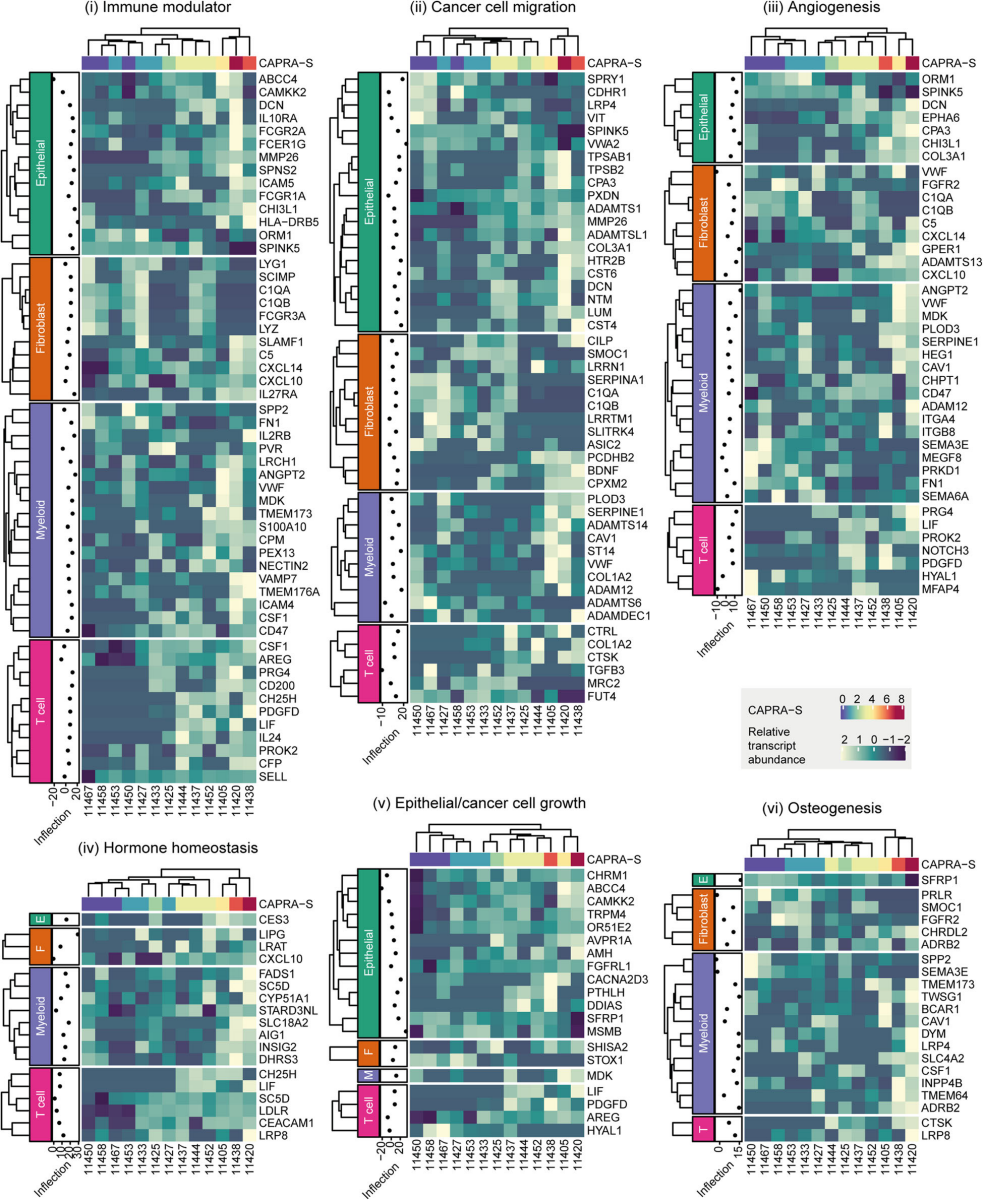
Recurrent functional categories identified in differentially transcribed secreted and transmembrane genes. The estimated inflection point for each gene shows the CAPRA-S risk score at which the transcriptional change was fastest; values < 0 or > 7 indicate an early or late change, respectively

In order to allow an in-depth interpretation of the concurrent transcriptional differences for cell-surface and secreted protein-coding genes across cell-types, we produced a cell-type and disease-specific annotation database integrating curated cell-specific Gene Ontology information [31] with more than 1500 scientific articles (Supplementary file 3). This database allowed us to identify six recurring hallmarks of cancer (Fig. 2): (i) immune modulation; (ii) cancer cell migration; (iii) angiogenesis; (iv) hormonal homeostasis; (v) epithelial/cancer cell growth; and (vi) osteogenesis. Among the immune modulation related genes, a balance exists between pro and anti-inflammatory. This balance appears to be dynamic along the disease progression course. The epithelial cell migration hallmark includes three main functional clusters: tissue remodelling, tissue fibrosis and direct epithelial-to-mesenchymal transition. The differential transcription events of those three classes do not appear to be concentrated on any particular stage of disease progression.

Similarly, angiogenesis signalling appears to be sustained along the whole disease progression. A cluster of genes linked to platelet recruitment and endothelial cell migration is differentially expressed in synergy by both myeloid and T cells. Several transcriptional alterations from both epithelial and immune cells were linked with hormonal and lipid homeostasis, a key molecular hallmark in prostate cancer [47]. Within this set, the most recurring metabolite that is linked with differentially transcribed genes is cholesterol. While all four cell types contributed similarly for most hallmarks, a clear bias is present for cancer cell growth, osteogenesis and hormone modulation, which genes are differentially transcribed in epithelial and immune cells types, respectively. As the most compelling signal, immune modulation was selected for further investigation.

### Immune modulation is associated with cancer grade and targets predominantly monocyte-derived cells

**Fig. 3:**
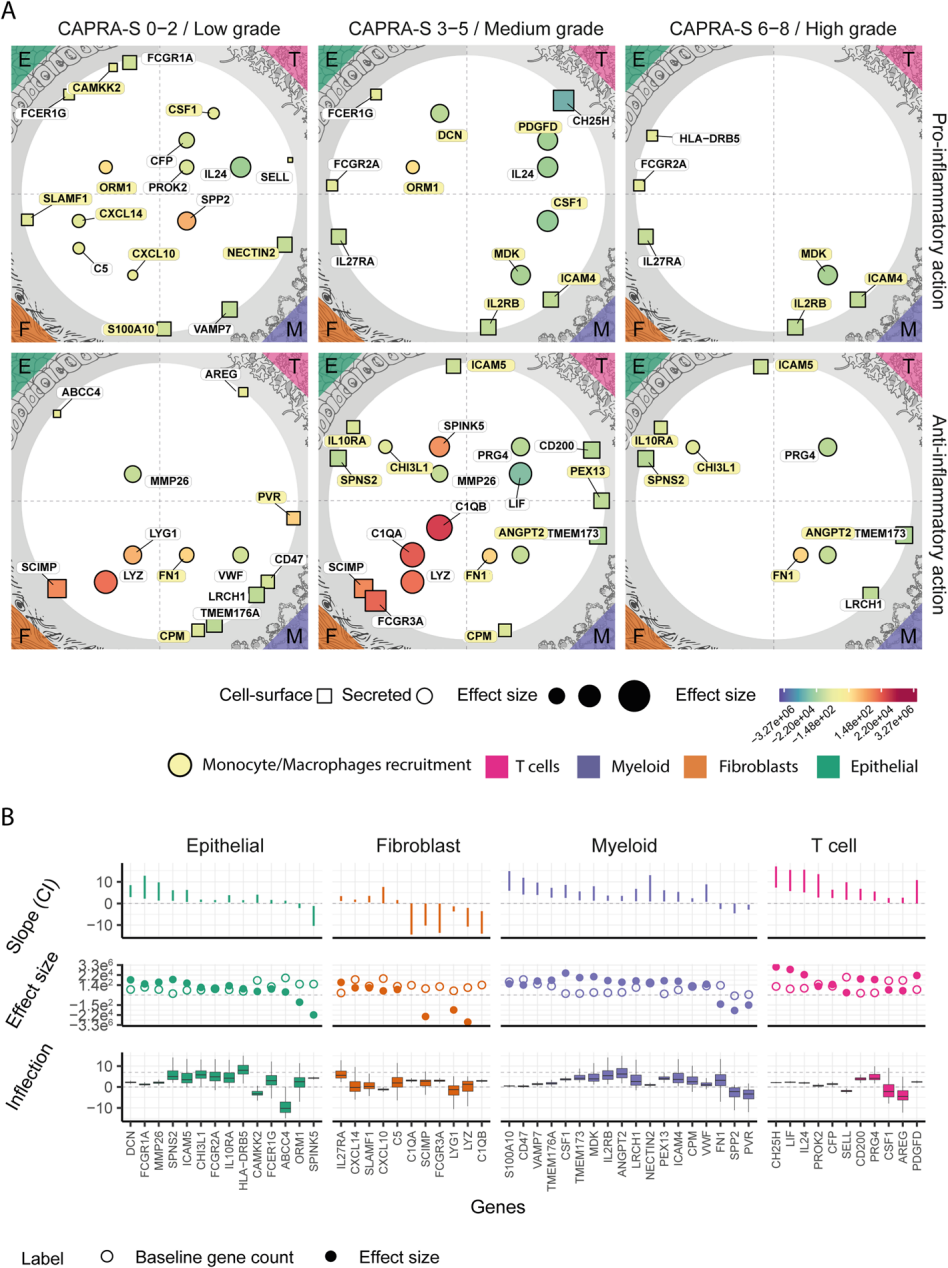
Multi cell-type immune-modulation changes with risk progression and is mainly targeted at monocyte-derived cells. The landscape of the immune-modulation related genes encoding cellular interface-proteins (i.e. cell-surface or secreted) inferred to be differentially transcribed across CAPRA-S risk scores, grouped by cell type. **A** Map of the secretory (represented as circles) and cell-surface (represented as squares) protein-coding genes that are differentially transcribed across the four cell types. The data point size is proportional to the baseline transcript abundance. The colour coding represents the effect size. Genes with a similar inflection point (i.e. at what stage of the disease a transcriptional change happens) are clustered vertically (CAPRA-S risk score < =2, > 2 and < =5 and > 5). Genes are split horizontally according to their pro- or anti-inflammatory role. Genes encoding for proteins that target monocyte-derived cells are highlighted in yellow. **B** Statistics of the differentially transcribed genes displayed in panel (**A**). Top: credible interval of the association between transcript abundance and CAPRA-S risk score. Middle: inferred effect size (full dots) and baseline transcription (empty dots). Bottom: credible interval of the CAPRA-S value for the transcriptomic change (i.e. inflection point; e.g., the gene HLA − DRB5 is upregulated in late stages of the disease)

In order to elucidate the role of the four cell types in the immune response to primary prostate cancer and their potential interactions, we focused on genes that encode for cell-surface and secretory proteins involved in immune modulation. In doing so, we again used the fitted inflection point of the sigmoid model to distinguish between early (i.e. low CAPRA-S risk score) and late (i.e. high) transcriptional changes. The balance between pro and anti-inflammatory signalling from the four cell types tracks the risk score covariate (Fig. 3). The number of differentially abundant gene-transcripts encoding for pro-inflammatory proteins remains roughly constant through the risk range, with 18 genes for CAPRA-S risk score ≤ 2 and 14 for CAPRA-S risk score > 2. On the contrary, the number of altered anti-inflammatory-related genes significantly expands (*p*-value 0.015; t-test) for more advanced stages of the disease, with 12 genes against 20 for the two risk score categories, respectively.

Overall, a large proportion (14 genes of 27) of the inflammatory-related transcriptional alterations across all four cell types is involved in the recruitment of monocytes and macrophages [48–55] (highlighted in yellow in Fig. 3a). These include CAMKK2 [56], ORM1 [57] and DCN [58, 59] in epithelial; IL2RB [60, 61], ICAM4 [62, 63], DCN [58, 59] and MDK [64, 65] in myeloid cells; and CSF1 [48] and PDGFD [66] in T cells. In addition, we identified a known fibroblast-macrophage chemotactic interaction including the regulation of the cytokines CXCL10 [51], CXCL14 [50] and the receptor SLAMF1 [52, 53] for fibroblasts; with COL1A2 [67] and CYR61 [68] (for CAPRA-S 6–8) altered in myeloid cells known to function as a co-stimulatory loop. A smaller cluster of genes was linked with T cell recruitment and inflammation, including CFP [69], IL24 [70], PROK2 [71], SELL [72]. Interestingly, epithelial cells upregulate a cluster of receptor genes normally involved in antigen recognition and presentation in immune cells [73], including an MHC class II cell surface receptor (i.e. HLA-DRB5) and three Fc receptors (i.e. FCER1G, FCGR1A and FCGR2A).

Overall, the significant genes associated with anti-inflammation across the four cell types targets a more heterogeneous set of cell types than the pro-inflammatory ones. Monocyte-derived cells are mainly targeted by genes that are differentially transcribed in epithelial and myeloid cells. These include the receptor genes SPNS2 [74, 75], IL10RA [76] and ICAM5 by epithelial cells; and the receptor genes CPM [77] and PEX13 [78] and the secreted protein genes FN1 [79] and ANGPT2 [80] by myeloid cells. Another cluster of genes targets predominantly T cells, including AREG [81, 82], CD200 [83], LRCH1 [84], CD47 [85]. Fibroblasts mainly downregulate pro-inflammatory cell-surface and secreted protein genes, such as FCGR3A and C1QA/B.

### Increased monocyte-derived cell infiltration in tumours is associated with lowered disease-free survival

In order to test the relevance of recruitment of monocyte-derived cells suggested by our integrated transcriptional analysis, we performed a differential tissue composition analysis (i.e. a test for difference in cell-type abundance between conditions) based on an independent methodology and independent patient cohort. We used a higher-order Bayesian inference model [34] that integrates deconvolution and downstream regression modules in a joint model (that showed superiority compared with the serial use of deconvolution and regression; Fig. S8) on an independent cohort of 134 patients from the primary prostate cancer.

**Fig. 4:**
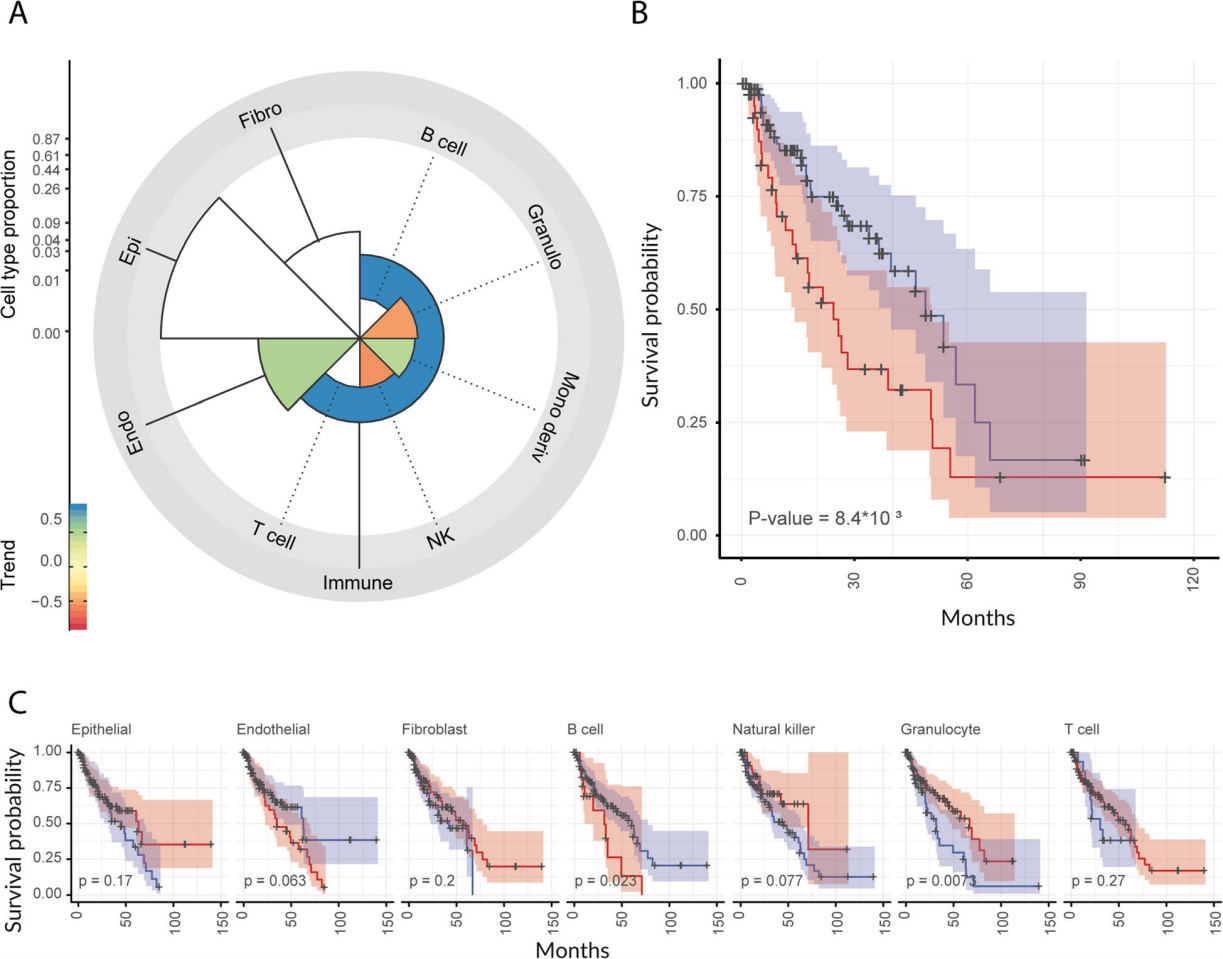
The abundance of monocyte-derived cells relative to total immune cells is positively associated with the CAPRA-S risk score and negatively associated with disease-free survival. Association of monocyte-derived cell abundance (see Materials and Methods) with disease-free survival in the independent primary prostate cancer TCGA dataset (*n* = 134). **A** Polar plot of differential tissue composition of primary prostate cancer TCGA samples for which CAPRA-S risk score information is available, with the factor of interest being CAPRA-S risk score. The y-axis (scaled by the fourth root) represents the overall cell type abundance; the colour coding reflects the association between cell type abundance and disease-free survival (coloured = significant association). **B** Kaplan–Meier plot of patients (*n* = 134) with low (blue) or high (red) monocyte-derived cell infiltration in the tumour specimen (proportion cut-off = 0.0048; see Materials and Methods section, Survival analyses subsection). **C** Kaplan–Meier plot for the other cell types included in the analysis

The Cancer Genome Atlas (TCGA) dataset [7] included both disease-free survival and CAPRA-S score information. The deconvolution module of this algorithm bases its supervised inference of cell-type proportions on a collection of 250 curated publicly available transcriptional profiles (including BLUEPRINT [35], ENCODE [36], GSE89442 [37] and GSE107011 [38]), encompassing 8 broad cell categories and 18 phenotypes. The deconvolution model uses those reference deconvolution signatures to estimate the contribution of each cell type to the observed mixed transcriptional signal (i.e. TCGA tissue RNA sequencing data). This analysis provides tissue composition estimates as well as their association with risk score [34]. Overall, we estimated a median of 88% for epithelial cellular fraction across samples (consistent with public literature [86], Fig. S5), 4.8% for endothelial, 4.8% for fibroblasts, and 1.6% for immune cells. The differential tissue composition analysis showed a significant positive association with the CAPRA-S risk score of the monocyte-derived and a negative association of the natural killer and granulocyte cells (95% credible interval excluding 0; Fig. 4a).

In order to test whether the enrichment in monocyte-derived cells is clinically relevant, we generated Kaplan–Meier curves using the estimated cell-type abundances. The stratification of patients based on the extent of monocyte-derived cells infiltration revealed a significant separation of the survival probabilities (Fig. 4b). For comparative purposes, we tested patient stratification for the other cell types included in the model. As a result, only granulocytes and B cells (with the poor outcome cohort including only a few patients) showed a significant negative association. In contrast, no other significant associations were detected for other cell types, including epithelial, endothelial, fibroblasts, and immune cell types, including T cells and natural killers (Fig. 4c). The negative association for the epithelial component might be due to the absence of the mesenchymal component in the epithelial deconvolution-signature derived from public data.

### Macrophage proximity to epithelial glandular clusters increases with tumour progression

In order to validate our findings and gather more in-depth knowledge about the role of macrophages in disease progression, we performed a spatial analysis at the single-cell level of 63 prostate biopsies from the independent RadBank cohort of 17 patients with localised prostate cancer, spanning a wide range of CAPRA risk scores (Table S1). Using 7 immuno-fluorescent dyes (including DAPI) with 6 others linked to antibodies (CD3, CD20, CD68, CD11c and HMWCK), cell size and shape, we were able to identify 6 major cell types: T cells, B cells, macrophages, dendritic myeloid, epithelial basal and stromal cells. Epithelial basal cells define prostate glands, where cancer cells originate and co-localise (confirmed by pathological evaluation). PDL1 expressing cells were also categorised using a PDL1-linked dye. Overall, we sampled an average of 41 × 10^3^ cells per biopsy (standard deviation 2.7 × 10^4^). The most abundant of the categorised cell types was epithelial basal (8.06% on average), followed by T cells (5.03% on average).

**Fig. 5:**
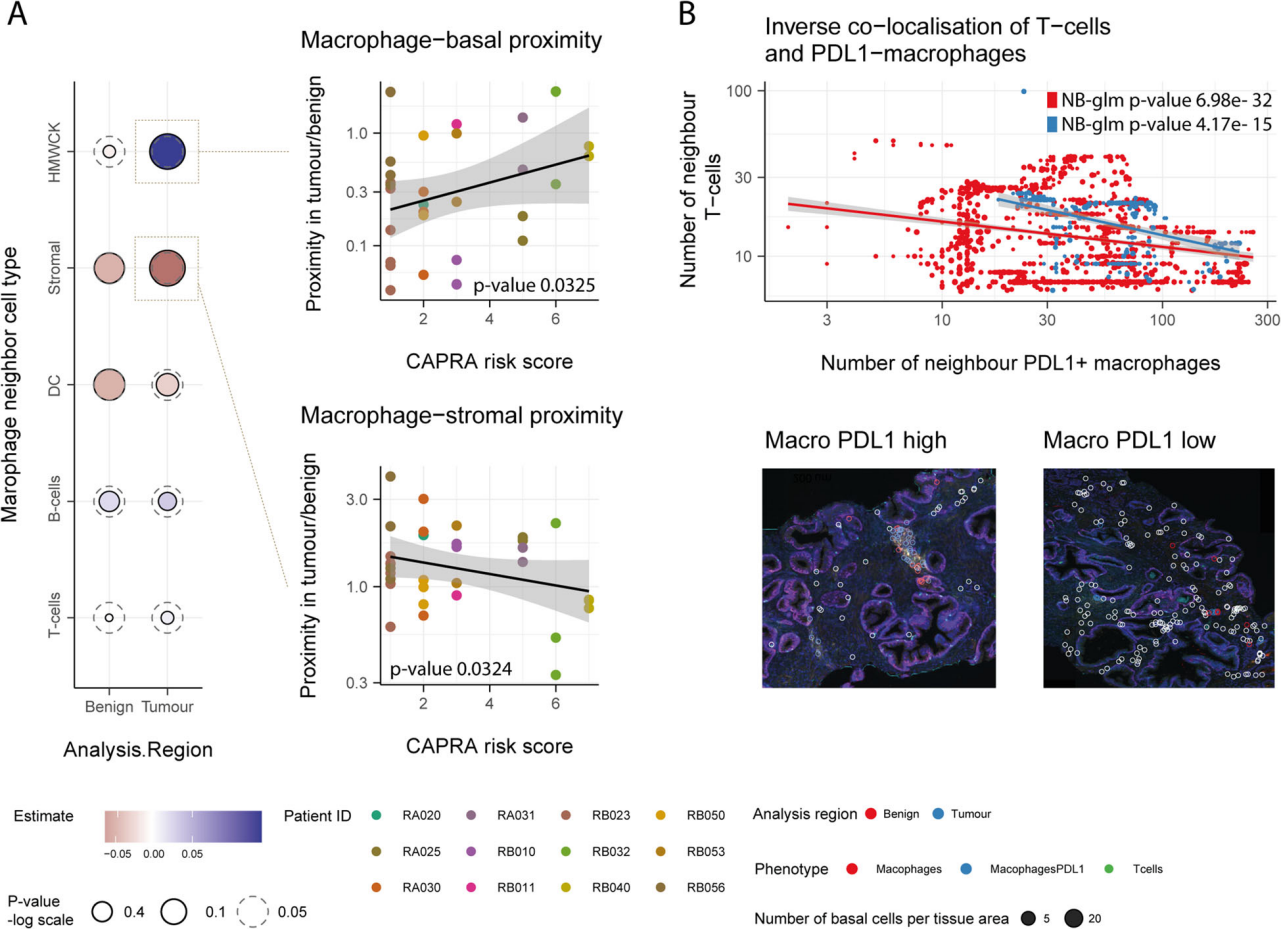
The analysis of multiplex-immunohistochemistry (*n* = 17) reveals proximity patterns of macrophages along disease progression. **A** Association between macrophage proximity and CAPRA risk score for five cell types identified from the multiplex immunohistochemistry. Proximity is calculated as the number of neighbour cells per tissue area and summarised using the median for each tumour biopsy. (left) Association between macrophages and epithelial basal cells (top) or stromal cells (bottom) and CAPRA risk score shown in panel (**A**). Only the 12 patients with both tumour and surrounding benign tissue are displayed (right). **B** Decreased proximity of T cells with epithelial basal in the presence of PDL1 expressing macrophages. The bottom section shows the multiplex immunohistochemistry tissue from patient RB010, with two examples of the presence (left) or absence (right) of PDL1 macrophages close to prostate glandulae. White circles surround the labelled T cells, blue and red circles surround macrophages which are PDL1 low and high, respectively

We estimated the association between macrophage proximity to five other cell types and CAPRA risk score (Fig. 5a). Across the five cell types, the average number of neighbour cells to macrophages ranges from 1.4 to 23.0. The strongest positive association was between macrophage and epithelial basal cells in tumour areas (*p*-value 0.0325; Fig. 5a-top-right), while the strongest negative association was for stromal cells in tumour areas (*p*-value 0.0324; Fig. 5a-bottom). Overall, the average proximity between macrophages and other cells, aggregated by biopsy, did not strongly associate with the CAPRA risk score. In order to gather evidence that the increased proximity of macrophages to the prostatic gland structures in advanced cancer stages had some direct effect on the local immune microenvironment, we tested the hypothesis that macrophages in proximity to gland structures would displace T cells. We observed an inverse association between the number of neighbour macrophage expressing PDL1 to epithelial basal cells and the number of T-cell neighbours (Fig. 5b). Epithelial basal cells that are close to clusters of PDL1 expressing macrophages tend to be further away from T cells.

## Discussion

To date, in-depth analyses of genomic features of prostate cancer alone, including single nucleotide variants and small and large structural rearrangements, have not been sufficient to provide transformative prognostic tools or unveil the full complexity of this disease. Non-malignant cells within the tumour microenvironment make an integral contribution to the mechanisms that cause cancer progression. They are often modulated by cancer cells toward pro-tumorigenic behaviours. In this study, we significantly improved the knowledge of the molecular landscape of the primary prostate tumour microenvironment, revealing concurrent transcriptional changes in epithelial, fibroblast, myeloid and T cells along the CAPRA-S risk score range.

We optimised a combined fluorescence-activated cell sorting and ultra-low-input RNA sequencing protocol, allowing us to obtain high-quality sequencing data from inputs down to 1000 cells. Such a strategy is of general utility as it enables studies of rare cell types from both fresh tissue cores and biopsies. In order to optimally detect changes in transcription along the CAPRA-S risk score, we developed a novel statistical inference method, TABI. This model permitted modelling transcript abundance natively on continuous factors of interest with a minimal number of parameters (*n* = 4), avoiding loss of information due to the dichotomisation of the risk score into low−/high-risk patient groups. As suggested by multidimensional-scaling plots and supported by our inference, transcriptional change events are indeed continuously distributed along the whole risk score range. This method is of broad utility in all cases where a continuous (or pseudo-continuous) factor of interest is present (e.g., risk score, time and chemical concentration) and a monotonic change in transcript abundance is of interest. Furthermore, the novel parametrisation of the generalised sigmoid function that TABI is based on can be extrapolated for a wide range of applications. On the contrary, publicly available statistical models for continuous regression of transcript abundance, such as for temporal RNA dynamics [87], cannot be used in the context of risk score. This is because they require a large biological replication, specific experimental design and are not constrained to monotonic trends, affecting practical interpretability.

The compilation of a curated cell-type-specific database of gene functions for cell-surface and secreted protein-coding transcripts enabled the detection of several recurrent hallmarks of prostate cancer, characterised by the involvement of multiple cell types. The most striking aspect to emerge was the large number of differentially abundant gene-transcripts linked to monocyte-derived cell recruitment and modulation. The association of monocyte-derived cell recruitment with increased risk score was reflected in an orthogonal differential tissue composition analysis on the extensive Cancer Genome Atlas (TCGA) independent patient cohort against CAPRA-S risk score through a differential tissue composition analysis. This analysis was enabled by a robust Bayesian inference model, able to transfer the uncertainty of the estimation of tissue composition for each sample to the linear model linking cell-type proportion (across samples) to clinical variables. This aspect is particularly relevant considering the substantial noise associated with the inference of tissue composition (i.e. deconvolution). To test the clinical significance of quantifying monocyte-derived cell numbers within the tumour mass besides the CAPRA-S risk score, we produced Kaplan-Meier estimates using the inferred cell-type proportions along progression-free survival on the TCGA cohort. For both analyses, we identified the strongest association with clinical variables being monocyte-derived cells. The infiltration of cell types such as monocyte-derived cell populations has previously been shown to be linked to the extent of proliferative inflammatory atrophy lesions, chronic prostatic inflammation and cancer gradehttps://paperpile.com/c/BQQ95X/GLf0R [88]. In prostate cancer, specific and overall survival analyses have identified an elevated monocyte count as an independent prognostic factor for poor outcomehttps://paperpile.com/c/BQQ95X/vyBlX+pxdwu+xaA8T+2mcuI [89–92]. Furthermore, the infiltration of tumour-associated macrophages in prostate needle biopsy specimens has been shown to have potential as a predictive factor for PSA failure or disease progression after hormonal therapyhttps://paperpile.com/c/BQQ95X/eaIrr [93].

In order to validate further our hypothesis and enrich our knowledge about the relation of macrophages with epithelial and a range of immune cells along disease progression, we used multiplex immune-histochemistry to determine the immune context at the single-cell level in an independent cohort. This data supports the hypothesis of a weakened relation of macrophages with stromal compartments and a strengthened association with epithelial glandular clusters along the disease progression spectrum; glandular clusters being generally colocalised with cancer cells in tumour tissues. This aspect becomes highly relevant as the glandular areas in both tumour and adjacent benign compartments rich in PDL1 macrophages are poorer in T cells. PDL1 expressing macrophages have been associated with their M2 wound-healing phenotypehttps://paperpile.com/c/BQQ95X/VvaW8 [94]. The relationship between PD-L1 expression in intratumoral macrophages and prognosis in cancer patients is still controversial. The two competing hypotheses are (i) PDL1 intratumoral macrophages lead to dysfunctional T cellshttps://paperpile.com/c/BQQ95X/2mglb [95] or (ii) not having significant effectshttps://paperpile.com/c/BQQ95X/BJESQ [96].

Nonetheless, the expression of PDL1 in macrophages has been shown to induce anti-inflammatory cytokines such as IL-10https://paperpile.com/c/BQQ95X/VvaW8 [94]. Although PDL1 in macrophages may primarily function as protection against induced cell death, our study supports the hypothesis that it may have the effect of inducing an anti-inflammatory, immune cold local microenvironment, with adverse effects on disease progression. Although the role of several immune cell types has been widely investigated in prostate cancer, the driving forces of immune modulation, clinically relevant for immunotherapy resilience, are still under investigation. From the myeloid compartment, the main focus has been myeloid-derived suppressor cellshttps://paperpile.com/c/BQQ95X/i6jgh [15], that have been investigated mainly through blood and in-vitro analyses [97, 98].

## Conclusion

There has been limited benefit observed in prostate cancer through the unselected use of novel immune checkpoint inhibitors based on T cell receptor blockade (e.g., PD-1, PD-L1 and CTLA-4) https://paperpile.com/c/BQQ95X/i6jgh [15]. Such failure may, in part, be driven by our limited understanding of the dynamic interplay between immune components of the microenvironment and tumour cells. This study provides a clear direction for further investigation into mechanisms of the immune system, monocyte-derived cells in particular, that contribute to disease progression; for example, through changing the hormonal and growth-factor homeostasis through a sustained inflammatory state. Furthermore, this study provides a novel and robust method for detecting monotonic changes in transcript abundance over a continuous factor of interest such as risk and time that has broad applicability to other research areas. The methodological advances and the novel findings presented in this study provide a research framework for improved immune interventions.

## Supplementary Information

**Additional file 1.**

**Additional file 2.**

**Additional file 3.**

**Additional file 4.**

**Additional file 5.**

## Data Availability

The code used to conduct the analyses is available at github.com/stemangiola under the following repositories: prostate-TME-N52–2019; TABI@v0.1.3; ARMET@v0.7.1. Sequence data was deposited at the European Genome-phenome Archive (EGA), hosted by the EBI and the CRG, under accession number EGAD00001004948.

## Abbreviations

TCGA: The Cancer Genome Atlas
PBS: Phosphate-buffered saline
