# Supplementary material for: Transcriptome sequencing and multi-plex imaging of prostate cancer microenvironment reveals a dominant role for monocytic cells in progression
Source: BMC Cancer. 2021 Jul 22;21:846. doi: 10.1186/s12885-021-08529-6 (PMC8296706; doi:10.1186/s12885-021-08529-6)
Supplement: Supplementary file 5 — Additional file 5. [file 12885_2021_8529_MOESM5_ESM.docx]

# Supplementary results

## Quality control

After trimming and filtering, the sequenced reads of all samples achieved a Phred quality score above 28. The sequencing output ranged from 70 million (for sample 2C; [Fig. S2](https://docs.google.com/document/d/15KOHNKzIrABk740Uj4VxF1RnakfC5HwZrT8q50h-UP4/edit#D2L_fig_ref_Descriptive%20statistics%20of%20sequencing%20output%20and%20mapping%20for%20each%20sample%20(n%20=%2052)%20grouped%20by%20cell%20type.)) to 1 million reads (for sample 4C; [Fig. S](https://docs.google.com/document/d/15KOHNKzIrABk740Uj4VxF1RnakfC5HwZrT8q50h-UP4/edit#D2L_fig_ref_Descriptive%20statistics%20of%20sequencing%20output%20and%20mapping%20for%20each%20sample%20(n%20=%2052)%20grouped%20by%20cell%20type.)2). Most samples (n = 41) had more than 80% of reads uniquely mapped to the hg38 reference genome, with an exonic rate in the range of 50 to 70%. Overall, myeloid samples were characterised by a lower sequencing and mapping coverage compared to the other three cell types.; For all samples, a positive association was observed between CAPRA-S risk score and (i) number of mapped reads, as well as (ii) number of mapped reads to exons ([Fig. S](https://docs.google.com/document/d/15KOHNKzIrABk740Uj4VxF1RnakfC5HwZrT8q50h-UP4/edit#D2L_fig_ref_Pair%20plot%20showing%20the%20relations%20among%20sequencing%20and%20mapping%20statistics,%20stratified%20by%20cell%20type%20and%20CAPRA-S%20risk%20score.)9). This association was partly due to an abundant residual bacterial and viral genomic content, especially for the surrounding benign and low-grade cancers ([Fig. S](https://docs.google.com/document/d/15KOHNKzIrABk740Uj4VxF1RnakfC5HwZrT8q50h-UP4/edit#D2L_fig_ref_Proportion%20of%20bacterial%20and%20viral%20sequences%20in%20each%20sample,%20and%20its%20association%20with%20CAPRA-S%20risk%20score,%20cohort%20across%20cell%20types.)9).

# Supplementary tables

| **Patient** | **ID Age** | **Serum PSA (ng/ml)** | **Gleason Grade** | **T-stage** | **CAPRA** | **N. biopsies** |
| --- | --- | --- | --- | --- | --- | --- |
| RA014 | 50-59 | 11 | 3 | 1c | 3 | 1 |
| RA020 | 60-69 | 7.9 | 3 | 2b | 2 | 1 |
| RA025 | 50-60 | 80 | 3 | 3b | 5 | 3 |
| RA030 | 70-79 | 8.5 | 3 | 2b | 2 | 4 |
| RA031 | 60-69 | 6.4 | 5 | 2a | 5 | 2 |
| RB010 | 60-69 | 20 | 3 | 2b | 3 | 3 |
| RB011 | 60-69 | 10.9 | 2 | 2a | 3 | 2 |
| RB023 | 60-69 | 3.7 | 3 | 1c | 1 | 7 |
| RB032 | 70-79 | 27.7 | 4 | 1c | 6 | 7 |
| RB037 | 70-79 | 7.2 | 5 | 2b | 5 | 5 |
| RB040 | 60-69 | 10.3 | 5 | 3b | 7 | 4 |
| RB050 | 60-69 | 7.3 | 2 | 1c | 2 | 4 |
| RB051 | 60-69 | 4.1 | 2 | 1c | 1 | 6 |
| RB052 | 70 | 6.1 | 4 | 2a | 4 | 4 |
| RB053 | 69 | 10.1 | 2 | 2b | 3 | 3 |
| RB056 | 66 | 4 | 3 | 1c | 1 | 7 |

**Table S1.** Summary of the clinical characteristics of the subjects included in the multiplex immunohistochemistry analysis of primary prostate tumour biopsies.

#

#

# Supplementary figures

#
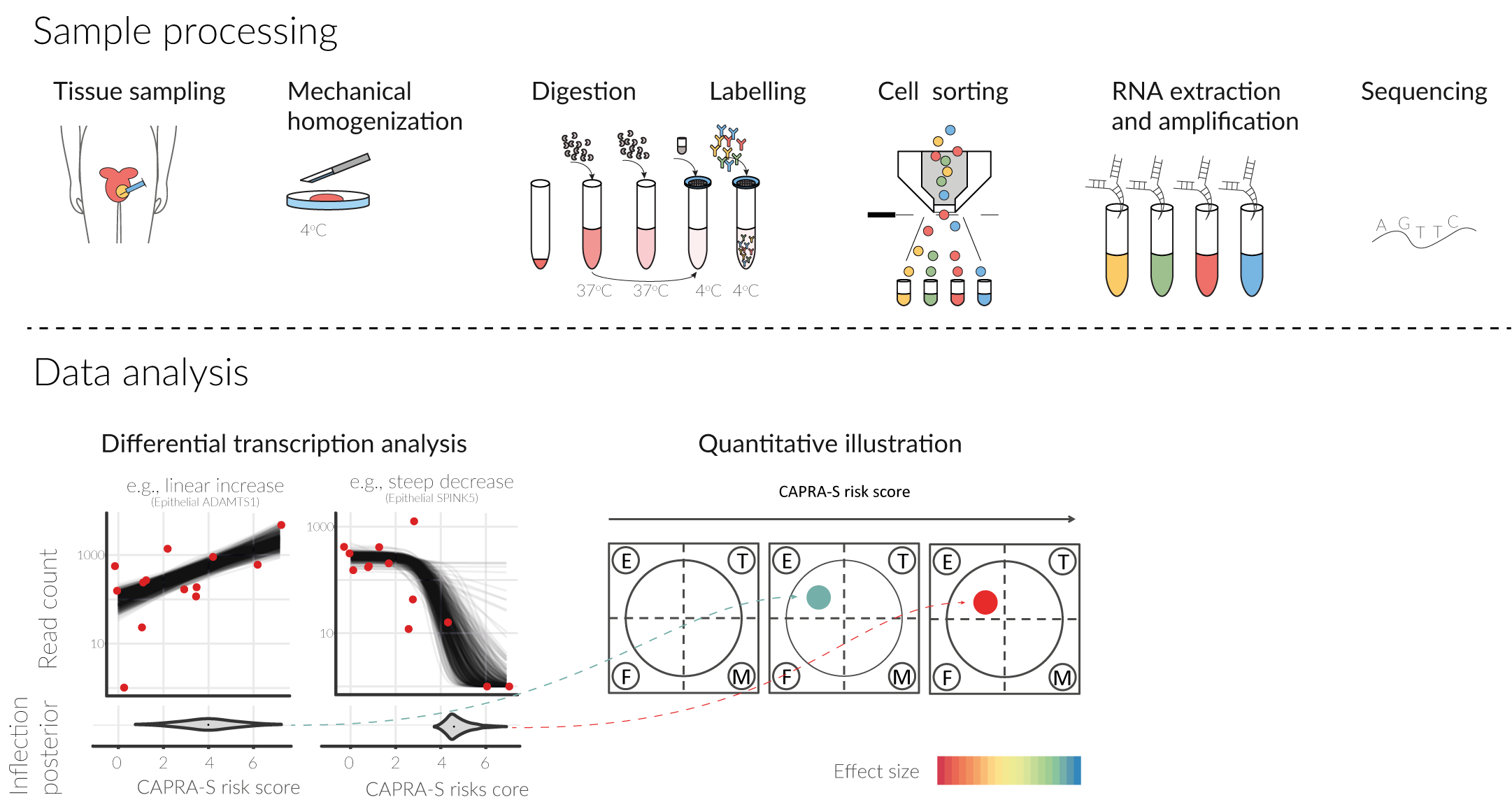


**Figure S1.** Diagram of the experimental and computational analysis pipeline.


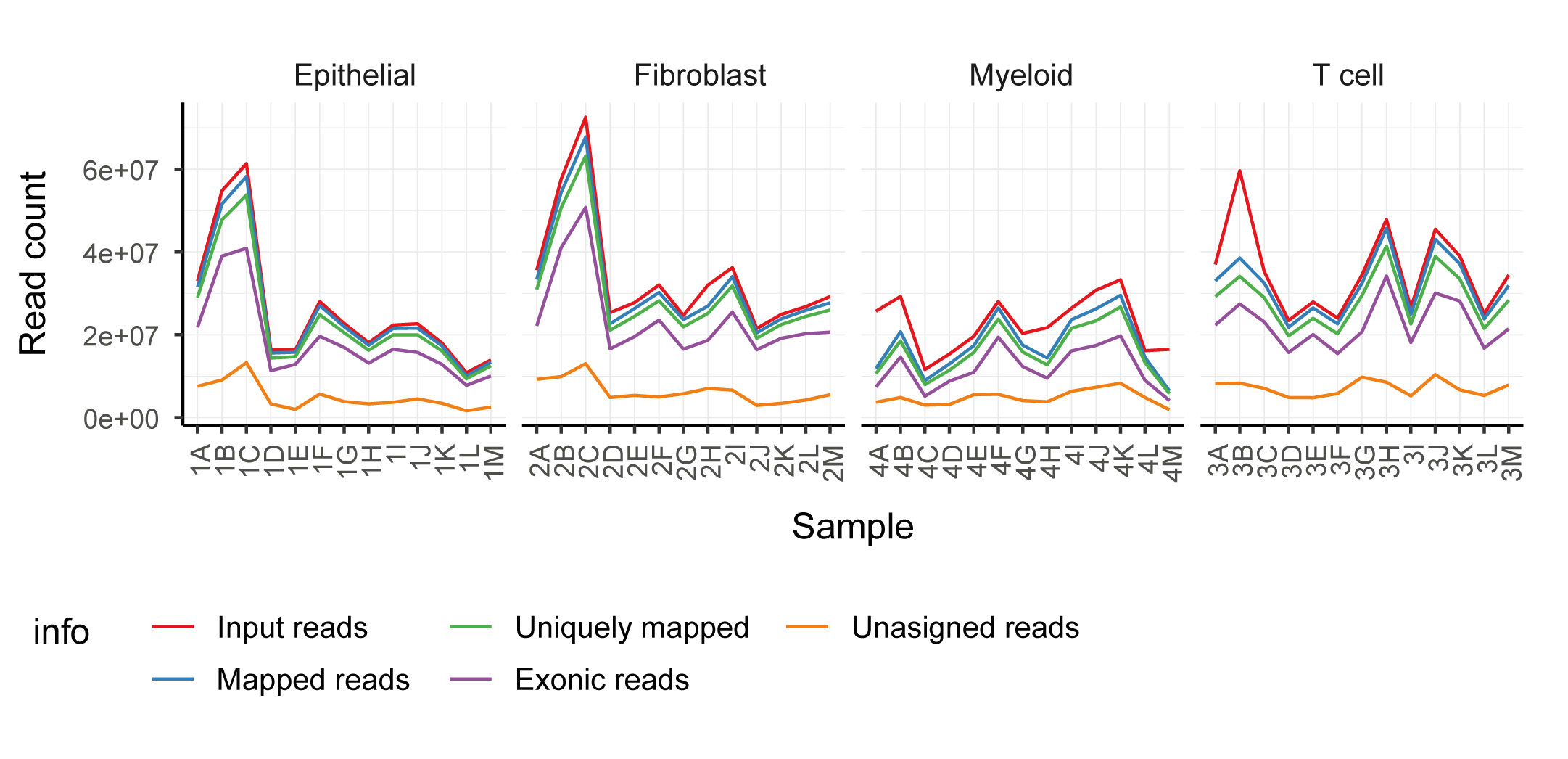


**Figure S2.** Mapping statistics for each sample, grouped by cell type.


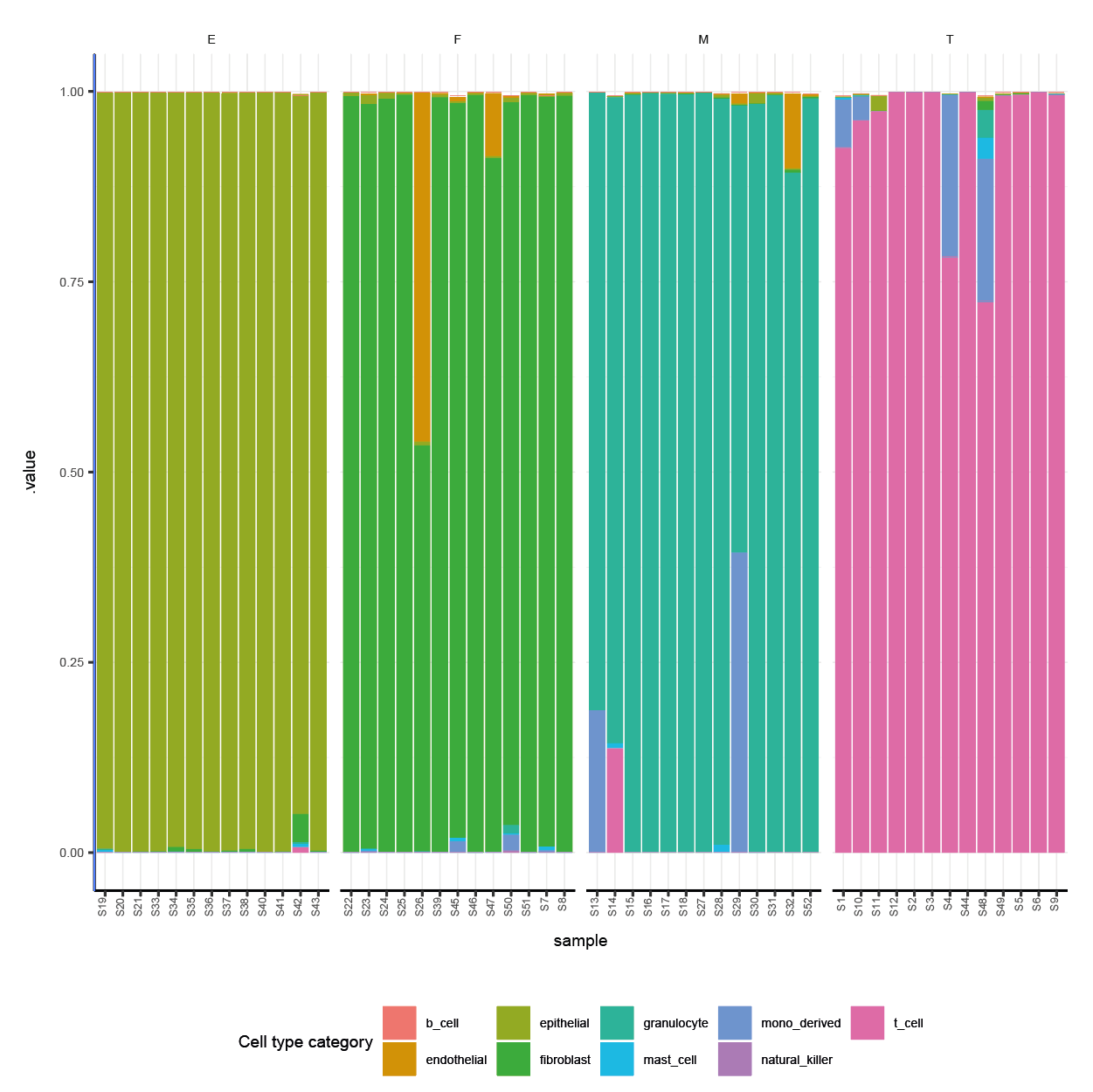


**Figure S3.** Bar-plot of cell-type composition for the four enriched cell types (E = epithelial; F= fibroblast; M = myeloid; T= T cell), Inferred by Bayesian inference deconvolution model.


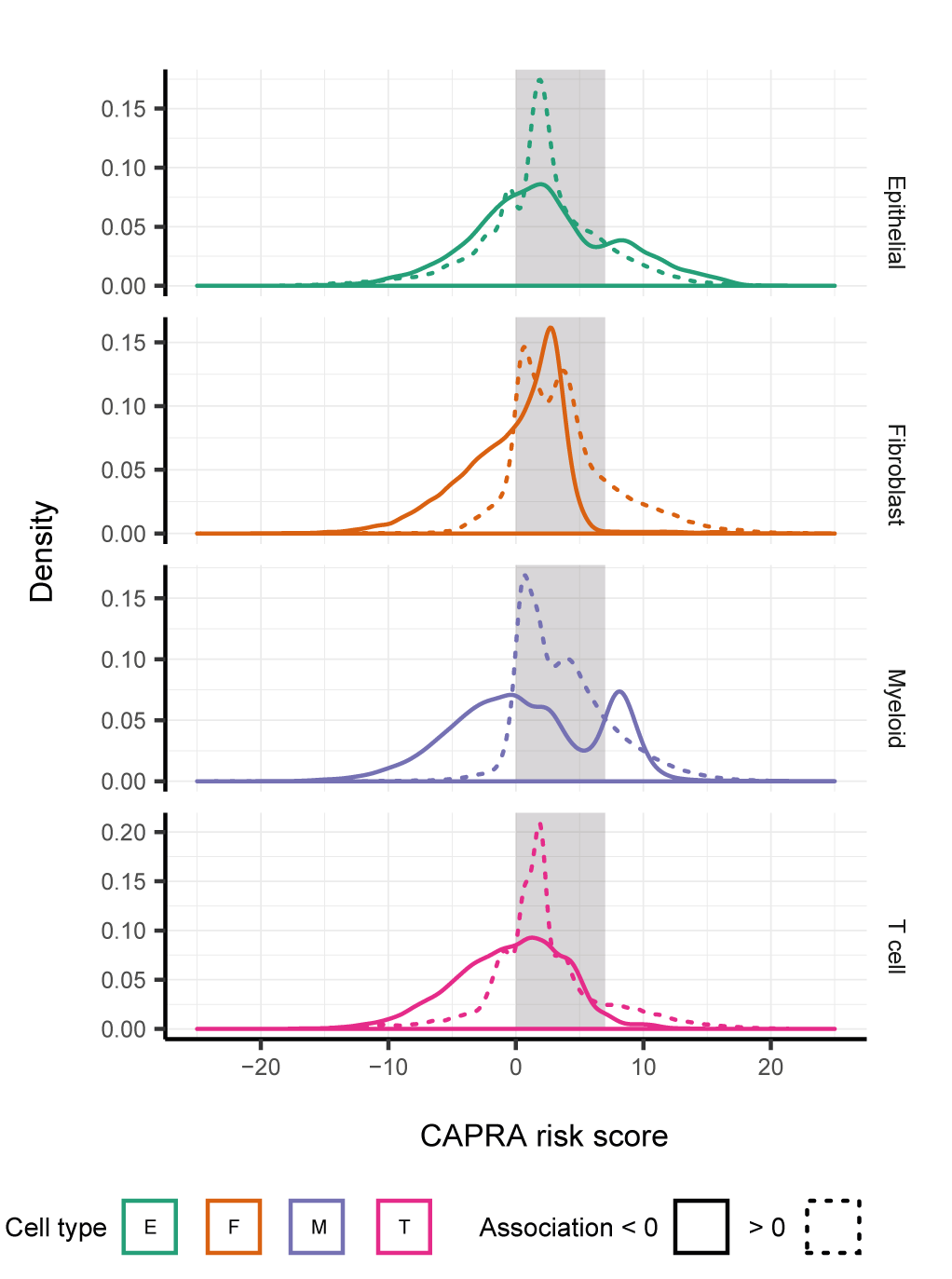


**Figure S4.** Distribution of the adjusted inflection points along the CAPRA-S risk score across cell types. The grey shade represents the range of CAPRA-S risk score in the 13-patient cohort. Inflection points outside that range represent exponential-like trends (either increasing or decreasing) that did not approach a plateau within the CAPRA-S risk score range.

**
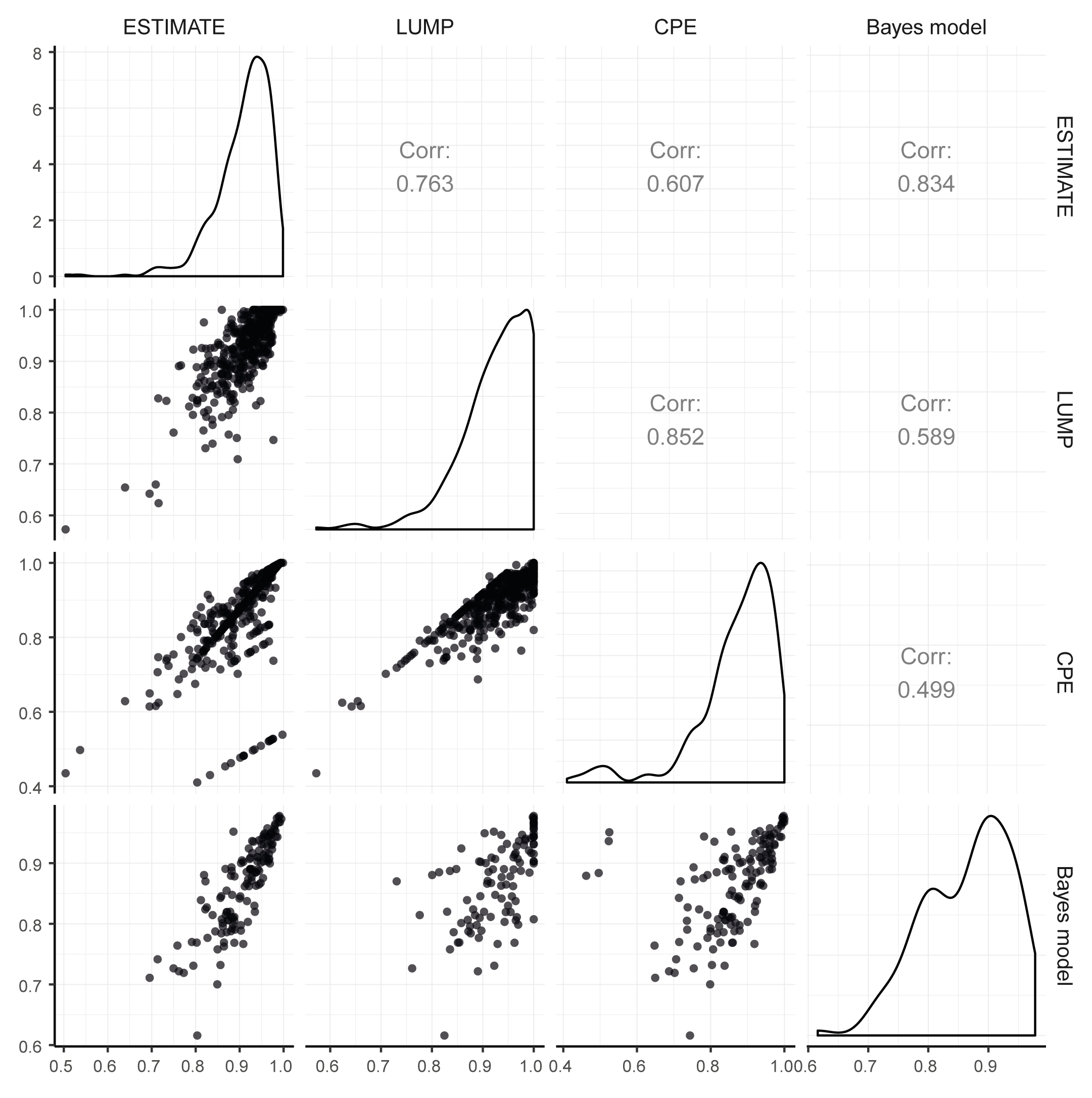
**

**Figure S5.** Pair plot showing the comparison between the inference of epithelial component in TCGA samples for our Bayesian inference model and gold standard tumour purity analysis[[1]](https://paperpile.com/c/kaRWXY/g8nFd).


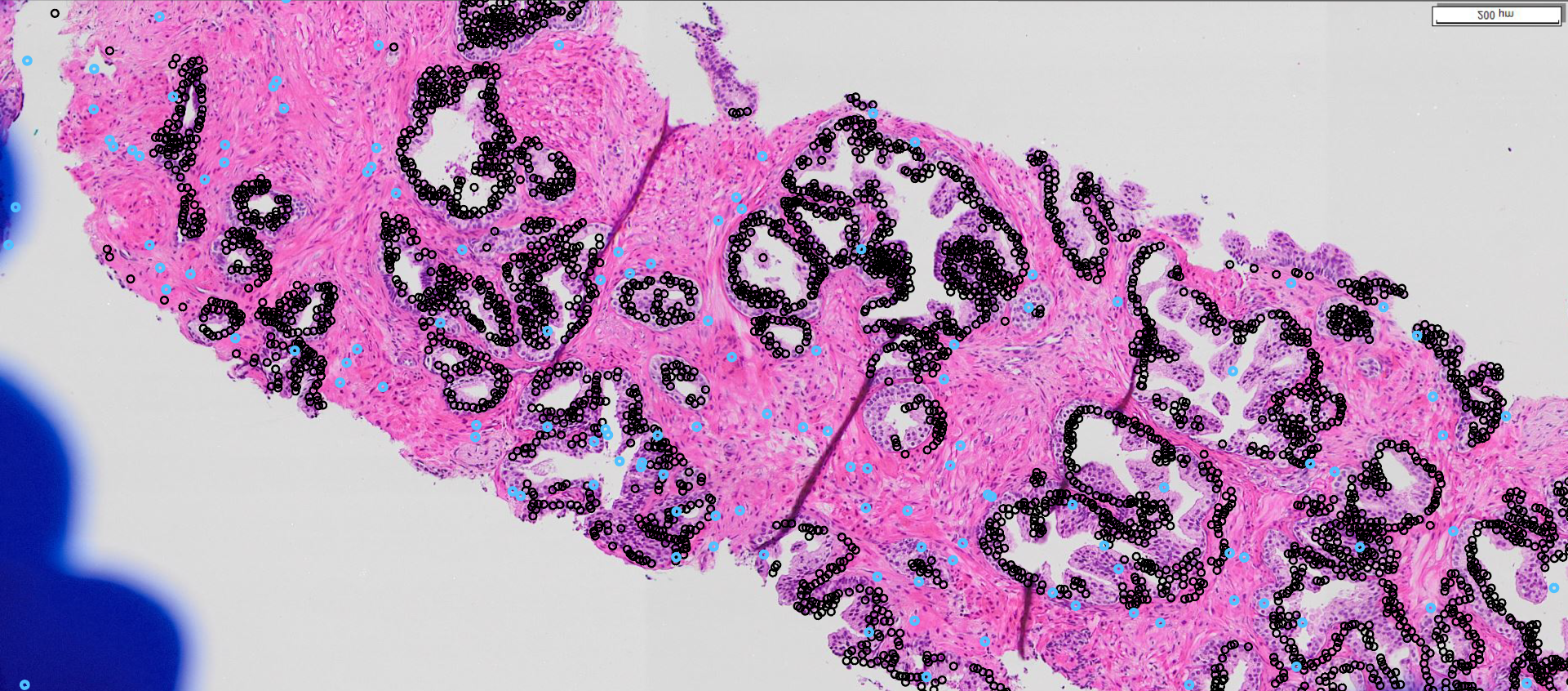


**Fig S6.** Example of stromal cells selection (blue circles). The black circles represent the HMWCK positive cells (epithelial basal). Stromal cells were labelled as negative for all markers, being DAPI positive, larger than units 70 and a highly elongated shape, with a ratio of largest dimension and smallest dimension > 2. These simple and stringent criteria lead to a highly specific but lowly sensitive selection. Specificity was prioritised over sensitivity considering the high number of stromal cells.


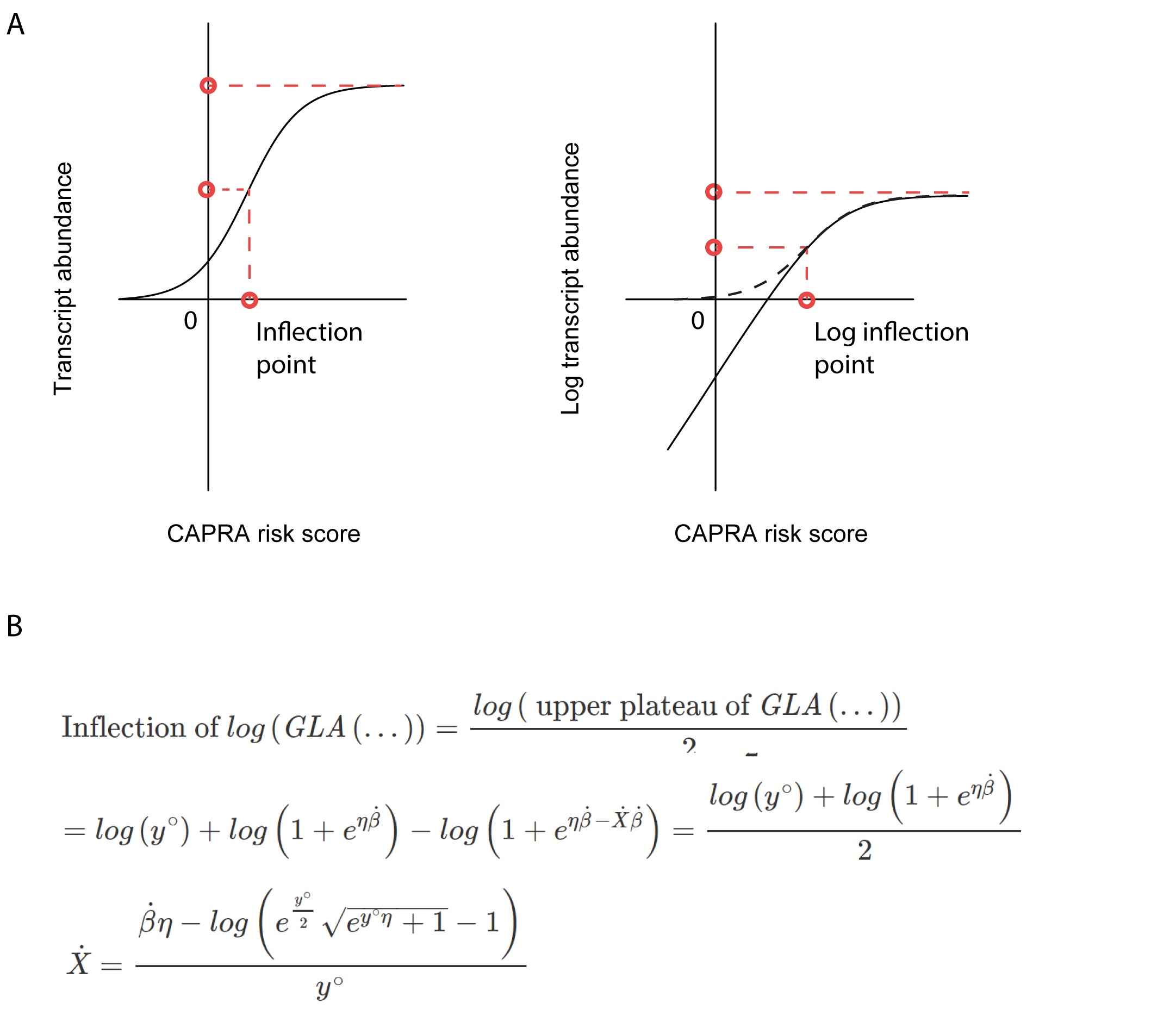


**Figure S7. A —** Illustration of the strategy for identifying the inflection point of the sigmoid function (left-panel) in log-scale (right-panel). The challenge is that to limit the number of parameters that the model needs, the lower plateau of the generalised sigmoid function was set to zero. Therefore, the inflection point of the logarithm transformed function is not defined (i.e. negative infinite). To define it, the lower plateau of the logarithm transformed function was also set to 0 (rather than -Inf). **B —** Formulae used to perform the transformation.

**
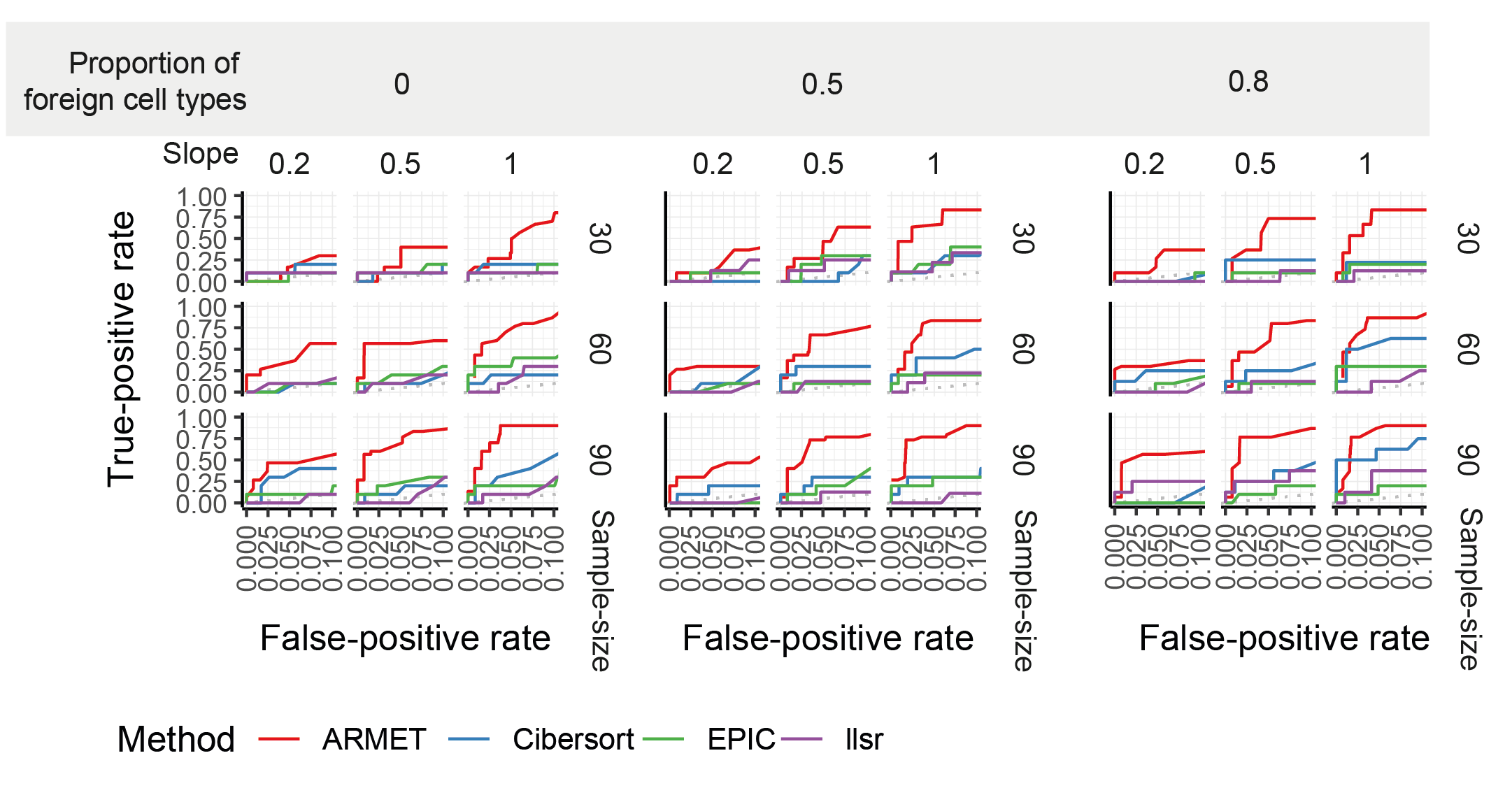
**

**Figure S8.** Benchmark of the accuracy of our higher-order differential tissue composition model against a combination of regression[[2]](https://paperpile.com/c/kaRWXY/JsLzR) with standalone deconvolution methods including Cibersort[[3]](https://paperpile.com/c/kaRWXY/T0Slg), EPIC[[4]](https://paperpile.com/c/kaRWXY/nXJmT) and llsr. Each quadrant shows receiving operating characteristic (ROC) curves for the 0-0.1 false-positive rate range. **Related Methods -** To test the accuracy of our higher-order method for the association analyses between deconvoluted cell-type abundances and a factor of interest (with a level of censoring in this benchmark, survival data), we composed in silico mixtures from single-cell RNA sequencing data (collection including 50,706 cells[[5]](https://paperpile.com/c/kaRWXY/lMUQx)^,^[^[6]^](https://paperpile.com/c/kaRWXY/jzTK3)^,^[^[7]^](https://paperpile.com/c/kaRWXY/JMHHY)^,^[^[8]^](https://paperpile.com/c/kaRWXY/zDdH9)^,^[^[9]^](https://paperpile.com/c/kaRWXY/7Wkh6)) labelled for cell-type identity using SingleR[[10]](https://paperpile.com/c/kaRWXY/3PHln)(monocyte-derived, CD8 and CD4 T-cells, B-cells and natural killer cells). The cell-type proportions used for building such mixtures were randomly generated from a Dirichlet distribution, following either an association (linear in the logit scale) with survival time (in log scale) for one of the five cell types or with no association (for four of the five cell types). The survival time was simulated from a gamma distribution fitted on real data (survival times of relapsed primary prostate cancer patients from the TCGA cohort), and for half of the simulated mixtures, the survival time was censored to 50% of its value (e.g. for 30 samples, 15 samples were censored; a survival time of 500 days was censored to 250). A total of 135 testing conditions were generated. These include a combination of the magnitude of change (slope; 0, 0.5 and 1), sample size (30, 60 and 90) and degree of missing information (percentage of cells foreign to our training data, neural cells[[11]](https://paperpile.com/c/kaRWXY/rSZBu)). The associations for the third-party algorithm combinations were estimated using three deconvolution procedures (Cibersort[[3]](https://paperpile.com/c/kaRWXY/T0Slg), EPIC[[4]](https://paperpile.com/c/kaRWXY/nXJmT) and llsr), which inferred cell-type proportions were fed to a Cox-regression algorithm[[2]](https://paperpile.com/c/kaRWXY/JsLzR) (with logit scaling). The slope of 0.2 in this benchmark corresponds to ~0.1-fold proportional change, which matches the magnitude of change we identify in primary prostate TCGA data. False-positive and true-positive rates were calculated along a range of significance thresholds, from 0 (none significant) to 1 (all significant).

[1. Aran D, Sirota M, Butte AJ. Systematic pan-cancer analysis of tumour purity. Nat Commun. 2015;6:8971.](http://paperpile.com/b/kaRWXY/g8nFd)

[2. Borgan �rnulf. Modeling Survival Data: Extending the Cox Model. Terry M. Therneau and Patricia M. Grambsch, Springer-Verlag, New York, 2000. No. of pages: xiii 350. Price: $69.95. ISBN 0-387-98784-3 [Internet]. Statistics in Medicine. 2001. p. 2053–4. Available from:](http://paperpile.com/b/kaRWXY/JsLzR) <http://dx.doi.org/10.1002/sim.956>

[3. Newman AM, Liu CL, Green MR, Gentles AJ, Feng W, Xu Y, et al. Robust enumeration of cell subsets from tissue expression profiles. Nat Methods. 2015;12:453–7.](http://paperpile.com/b/kaRWXY/T0Slg)

[4. Racle J, Gfeller D. EPIC: A Tool to Estimate the Proportions of Different Cell Types from Bulk Gene Expression Data. Methods Mol Biol. 2020;2120:233–48.](http://paperpile.com/b/kaRWXY/nXJmT)

[5. Freytag S, Tian L, Lönnstedt I, Ng M, Bahlo M. Comparison of clustering tools in R for medium-sized 10x Genomics single-cell RNA-sequencing data. F1000Res. 2018;7:1297.](http://paperpile.com/b/kaRWXY/lMUQx)

[6. Cai Y, Dai Y, Wang Y, Yang Q, Guo J, Wei C, et al. Single-cell transcriptomics of blood reveals a natural killer cell subset depletion in tuberculosis. EBioMedicine. 2020;53:102686.](http://paperpile.com/b/kaRWXY/jzTK3)

[7. Ding J, Adiconis X, Simmons SK, Kowalczyk MS, Hession CC, Marjanovic ND, et al. Systematic comparative analysis of single cell RNA-sequencing methods [Internet]. 2019 [cited 2020 Oct 18]. p. 632216. Available from:](http://paperpile.com/b/kaRWXY/JMHHY) <https://www.biorxiv.org/content/10.1101/632216v1>

[8. Karagiannis TT, Cleary JP Jr, Gok B, Henderson AJ, Martin NG, Yajima M, et al. Single cell transcriptomics reveals opioid usage evokes widespread suppression of antiviral gene program. Nat Commun. 2020;11:2611.](http://paperpile.com/b/kaRWXY/zDdH9)

[9. Official 10x Genomics Support [Internet]. [cited 2020 Oct 26]. Available from:](http://paperpile.com/b/kaRWXY/7Wkh6) <http://support.10xgenomics.com/>

[10. Aran D, Looney AP, Liu L, Wu E, Fong V, Hsu A, et al. Reference-based analysis of lung single-cell sequencing reveals a transitional profibrotic macrophage. Nat Immunol. 2019;20:163–72.](http://paperpile.com/b/kaRWXY/3PHln)

[11. ENCODE Project Consortium. An integrated encyclopedia of DNA elements in the human genome. Nature. 2012;489:57–74.](http://paperpile.com/b/kaRWXY/rSZBu)
